# Bioinspired Ionochromic Neuromorphic Transistors for Robotic Intelligent Perception

**DOI:** 10.1002/advs.76683

**Published:** 2026-07-24

**Authors:** Quanxing Yao, Xiaojian Zhu, Runsheng Gao, Qian Jiang, Cui Sun, Yi Du, Xuerong Liu, Lixun Wang, Haolong Li, Yuejun Zhang, Run‐Wei Li

**Affiliations:** ^1^ Zhejiang Key Laboratory of Magnetic Materials and Applications CAS Ningbo Institute of Materials Technology & Engineering Ningbo China; ^2^ Center of Materials Science and Optoelectronics Engineering University of Chinese Academy of Sciences Beijing China; ^3^ University of Chinese Academy of Sciences Beijing China; ^4^ Faculty of Electrical Engineering and Computer Science Ningbo University Ningbo China; ^5^ Eastern Institute of Technology Ningbo China

**Keywords:** human–machine interaction, ion doping, ionochromic transistor, tactile‐visual conversion

## Abstract

Intelligent perception with closed‐loop information acquisition, processing, and feedback is critical for humanoid robots and embodied intelligence systems. Ionochromic transistors hold great potential for on‐site signal processing and visual feedback. Here, we report a bioinspired ionochromic neuromorphic device with integrated signal‐processing capabilities for intelligent perception and display. The transistor unit consisting of poly(3‐hexylthiophene) (P3HT) and [EMIM][TFSI] (1‐ethyl‐3‐methylimidazolium bis(trifluoromethylsulfonyl)imide) ion gel, achieves synchronous conductance modulation and reversible color change via voltage‐controlled ion doping effects, mimicking biological synaptic response, and color regulation in chameleons. This dual‐functional behavior originates from the generation of polarons/bipolarons that reconfigure the P3HT energy levels and modify optical transitions. The device exhibits a high‐contrast electrochromic transition, with absorbance at 520 nm decreasing from 36.3% to 18.9%. Furthermore, a uniform electrolyte‐gated transistors array enables tactile signal visualization, verified by a visual Morse code system and robotic hand integration, realizing in situ tactile perception and accurate object recognition. This work provides an alternative solution for integrated intelligent perception, advancing the development of next‐generation human–machine interaction (HMI) platforms.

## Graphical abstract

A bioinspired ionochromic neuromorphic transistor couples synaptic conductance modulation with reversible color change through voltage‐controlled ion doping in P3HT/ion gel, mimicking biological synaptic plasticity, and chameleon‐like color regulation. Integrated with a robotic hand, the electrolyte‐gated transistor array visualizes tactile signals, and enables accurate object recognition, advancing integrated intelligent perception systems.

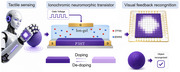

## 1 Introduction

Intelligent perception serves as the core function bridging humans and intelligent systems, playing a pivotal role in the development of humanoid robots and embodied intelligence equipment [1, 2, 3]. Closed‐loop perception, information processing, and feedback are critical to enhancing the efficiency and intuitiveness of such intelligent systems [4, 5, 6, 7]. For instance, tactile perception requires robots to acquire signals (e.g., pressure, contact position), process these signals, and subsequently provide real‐time feedback to support decision‐making for further operations [8]. To date, robots rely on various sensors for information acquisition. During this process, the collected signals are transmitted to a central processor for post‐hoc interpretation, which necessitates additional components such as displays for feedback. This isolated processing paradigm is unfavorable for fast, low‐power, and reliable closed‐loop signal processing, especially in array systems handling massively parallel signals [9, 10, 11, 12, 13, 14, 15]. In biological systems, such as chameleons, the skin integrates information acquisition, processing, and color change, enabling efficient environmental perception, and responsive color adjustments [16, 17, 18]. This integration not only reduces structural complexity and signal latency but also enhances intuitiveness. Therefore, developing bioinspired intelligent ionochromic devices provides a viable alternative for perception processing in robotic systems.

Electrolyte‐gated transistors (EGTs) are promising candidates for neuromorphic perception and information processing applications. As three‐terminal devices, EGTs can effectively mimic the signal memory and processing capabilities of biological synapses [19, 20, 21, 22]. Under the drive of gate voltage, ions migrate within the electrolyte to dynamically dope the channel, thereby modulating carrier concentration and enabling continuous, reversible tuning of channel conductance [23]. This gradual conductance modulation reproduces essential synaptic functions (e.g., short‐term plasticity (STP), long‐term plasticity (LTP)), allowing single EGTs to act as neuromorphic devices for learning and memory [24, 25, 26]. EGT‐based circuits have demonstrated excellent reliability and reproducibility in intelligent perception and edge computing scenarios (e.g., real‐time gesture recognition, robotic manipulation) and enable robots to adapt to dynamic environments through multimodal sensory and feedback integration [27, 28, 29, 30, 31]. Recent studies have shown that integrating electrochromic materials into EGTs can effectively couple electrical processing with on‐site color change to facilitate signal visualization [32]. Electrochromic materials reversibly modulate optical absorption through electrically driven redox reactions, directly converting electrical signals into color changes for synchronous visualization without the need for additional components [33, 34, 35]. Additionally, these materials exhibit tunable optical transitions, fast redox kinetics, and strong ionic‐electronic transport coupling, enabling rapid, low‐voltage optical modulation [36, 37]. Consequently, such devices hold great potential for unified perception‐computation integration and intelligent interaction applications.

**FIGURE 1:**
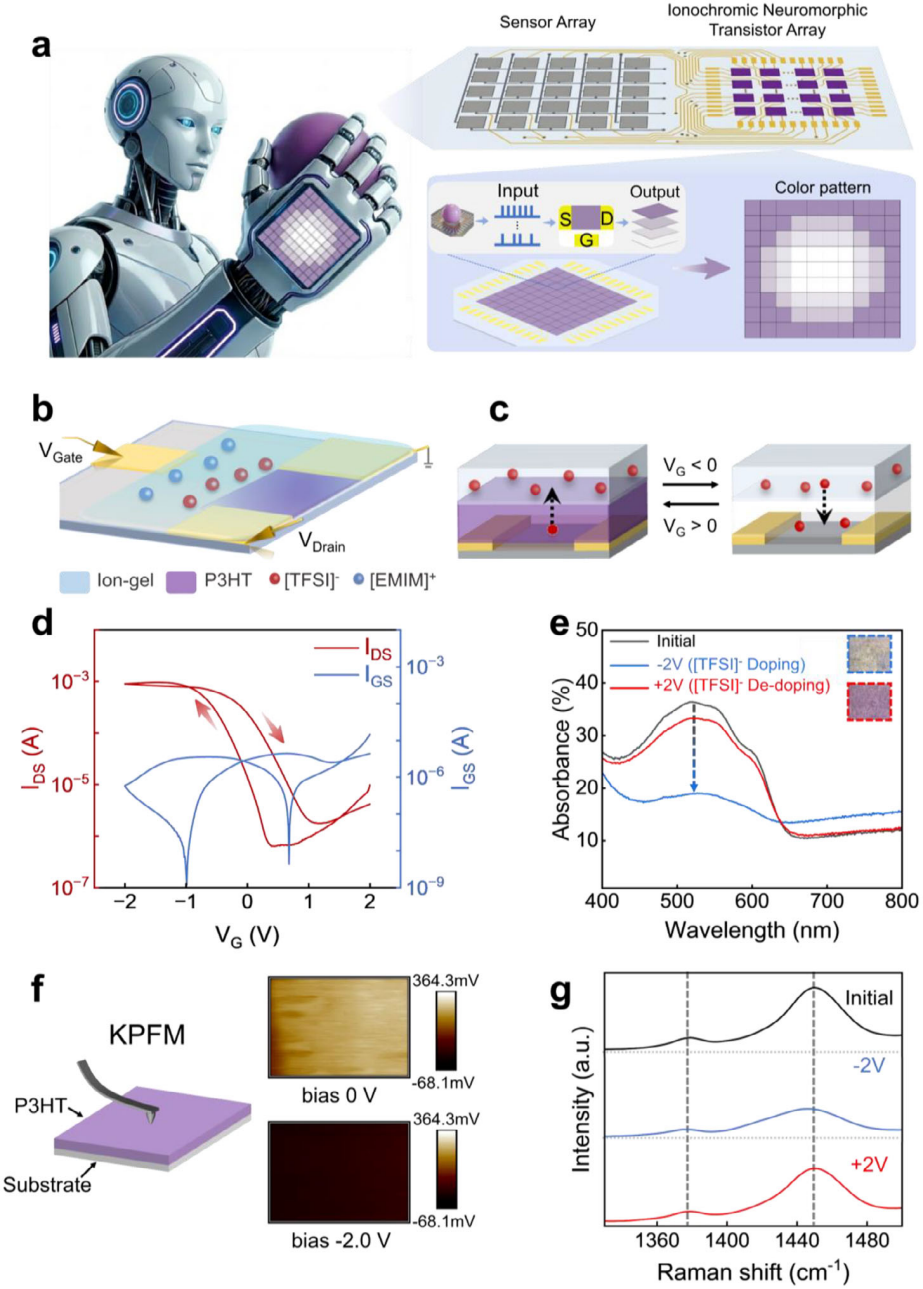
Tactile signal visualization in robotic perception systems. (a) Schematic illustration of tactile signal visualization achieved by integrating a tactile sensor array with an ionochromic neuromorphic transistor array. (b) Device architecture of the proposed P3HT‐based ionochromic neuromorphic transistor. (c) Schematic depiction of reversible ionochromic modulation driven by different biases, corresponding to ion doping and de‐doping within the P3HT channel. (d) Transfer characteristics of the ionochromic neuromorphic transistor. (e) UV–vis absorbance spectra and inserted optical images of the P3HT film under various voltages. (f) In situ Kelvin probe force microscopy (KPFM) surface potential maps of the P3HT film in the initial state (0 V) and under a −2 V. (g) Raman spectra of the P3HT film under various voltages.

In this work, we report the neuromorphic transistors with inherent ionochromic capabilities that can be integrated with tactile sensors for intelligent perception and display. Based on poly(3‐hexylthiophene) (P3HT) and ion gel, the proposed device exhibits reliable conductance modulation and color change through voltage‐controlled [TFSI]^−^ ion doping. These devices demonstrate typical synaptic characteristics along with stable, reversible ionochromic transitions. A uniform EGT array enables precise spatial visualization of tactile signals, which is verified via a visual Morse code typing system that translates pressure pulses into corresponding ionochromic changes. The robotic hand integrated with the sensor‐EGT device can in situ perceive tactile signals and convert them into visualizable ionochromic patterns, which can be further classified for object recognition (Figure 1a). This work offers a viable strategy for integrated intelligent perception‐feedback using advanced neuromorphic EGTs, promoting the development of compact tactile‐visual interactive platforms for next‐generation HMI systems.

## 2 Results and Discussion

### 2.1 Electro‐Optical Properties of Ionochromic Neuromorphic Transistor

The EGT transistor (Figure 1b) consists of an ion‐gel gate dielectric containing [EMIM][TFSI] ionic liquid, a poly(3‐hexylthiophene) (P3HT) organic semiconductor channel, and Au electrodes. The device fabrication process is detailed in Figure S1. As illustrated in Figure 1c, under a negative gate bias, [TFSI]^−^ anions within the ion gel migrate toward the P3HT channel and induce hole doping. Conversely, application of a positive gate voltage extracts these anions, restoring the P3HT film toward its neutral state.

To experimentally verify the voltage‐controlled electrical and optical properties of the as‐prepared EGT device, gate‐voltage‐dependent characteristics were systematically measured. Figure 1d exhibits distinct hysteresis in the drain current (*I_D_*) characteristics when gate voltage (*V_G_*) is swept from 2.0 to −2.0 V, achieving a conductance transformation of 10^3^, which confirms a pronounced conductance modulation effect. Simultaneously, the P3HT film undergoes a distinct electrochromic transition from its initial purple state to a nearly colorless, highly transparent state (insets of Figure 1e), demonstrating efficient modulation of the film's optical properties via electrically driven ion doping. Ultraviolet–visible (UV–vis) spectroscopy was employed to analyze the optical evolution, as shown in Figure 1e. The spectra reveal that voltage sweeping from 0 to −2.0 V leads to a marked reduction in the absorption peak within the 400–600 nm range, with the absorbance intensity at 520 nm decreasing from 36.3% to 18.9%. Reversing the bias to 2.0 V restores the spectrum to its initial profile and recovers the original color of the film, confirming the high reversibility of ionic gating.

Kelvin probe force microscopy (KPFM) was utilized to probe the ion‐doping process by mapping surface potential variations associated with ionic distribution. As shown in Figure 1f, the average surface potential of the P3HT film decreases from 360 mV in the initial state (0 V) to −60 mV under a −2 V bias, confirming [TFSI]^−^ anion doping at the channel interface. Raman spectroscopy was further employed to fully elucidate the molecular origins underlying the observed changes in conductance and absorption during the doping process. Figure 1g presents the Raman spectra of the P3HT film in the initial state, and under −2.0 V and 2.0 V, respectively. In the initial state, two characteristic bands are observed at 1450.6 and 1379.2 cm^−1^, corresponding to C═C and C─C stretching vibrations, respectively. Upon application of V_G_ = −2 V, these two peaks redshift to 1445.6 and 1374.2 cm^−1^. This phenomenon arises from electrochemical doping, where the *π*‐conjugated backbone of P3HT transitions from a benzoid‐like to a quinoid‐like configuration [32] (Figure S4). This structural transformation facilitates the generation of polarons and/or bipolarons, which narrows the *π*–*π* ^*^ band gap and modifies the main optical transitions, as illustrated in the energy level diagram (Figure S5). These well‐defined spectral results confirm efficient and reversible anion insertion into the P3HT channel under bias, which is essential for stable electrochromic behavior and conductance modulation in the synaptic transistor.

**FIGURE 2:**
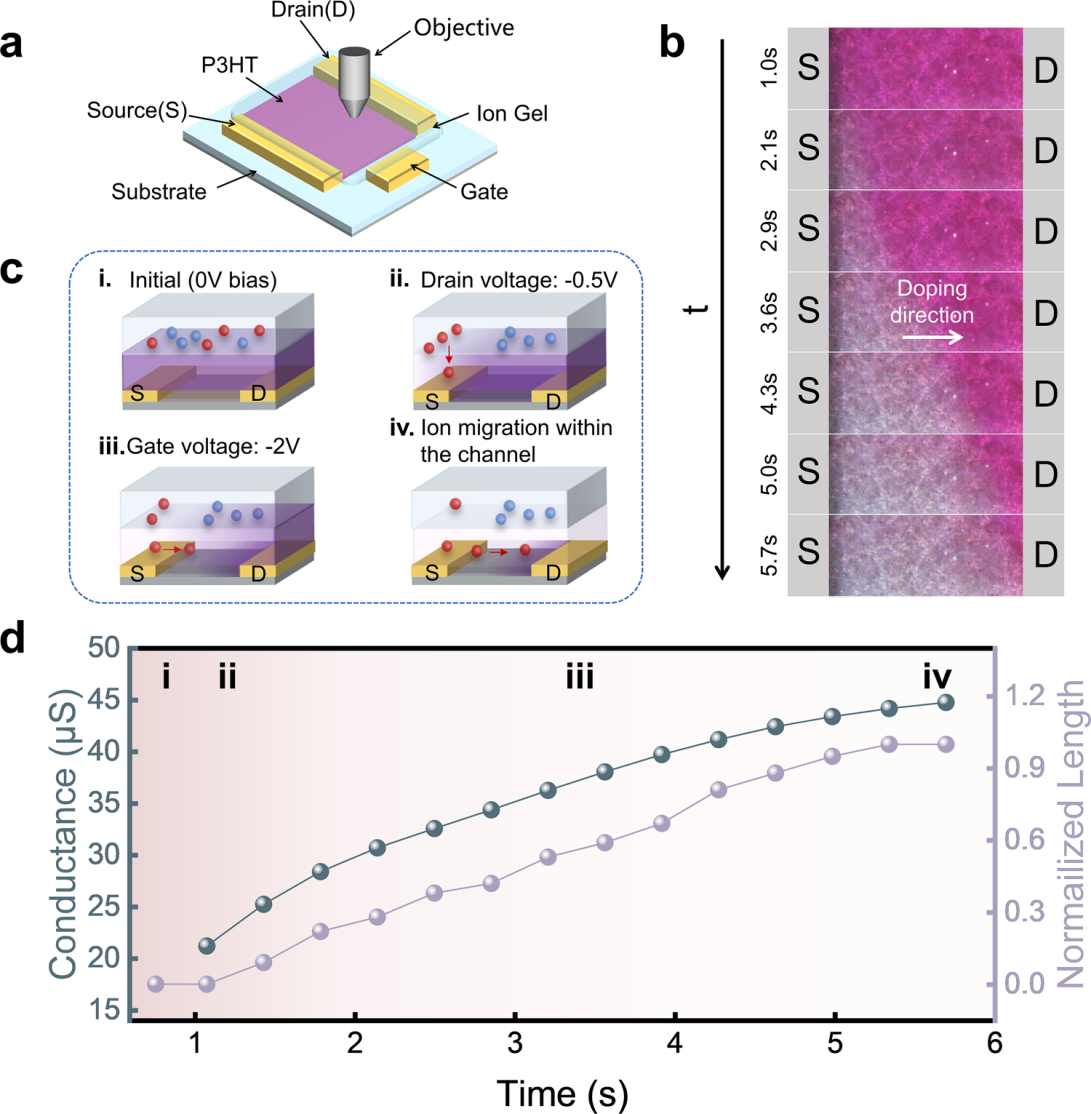
Spatiotemporal evolution of ion doping and synergistic electro‐optical modulation. (a) Schematic of the experimental setup for electrical and optical characterization. (b) Optical microscopy snapshots extracted from a video sequence showing the dynamic color evolution, where the arrow denotes the doping direction. (c) Schematic representation of ion migration and electrochemical doping within the device across four successive states (states i–iv): (i) initial state under 0 V bias; (ii) application of a −0.5 V drain voltage (*V_D_)*; (iii) application of a −2.0 V gate voltage (*V_G_*); and (iv) continuous ion migration within the channel. (d) Concurrent recording of channel conductance and normalized ionochromic length under a constant gate bias of −3.0 V and a fixed drain‐source voltage (*V_D_*) of −0.5 V.

Furthermore, in situ optical characterization was performed to monitor the continuous state evolution of the P3HT channel (Figure 2a) and resolve the spatiotemporal dynamics of ion doping. The results show that the ionochromic process initiates from the source side and gradually extends to the drain side, with uniform and smooth transitions indicating stepwise doping of the P3HT layer. The recorded optical images (Figure 2b) exhibit a distinct doping front propagating from the source to the drain terminals. This lateral propagation is driven by the synergistic effect of the drain and gate potentials, and the ionochromic evolution exhibits a clear linear dependence on time. This process essentially corresponds to the electrically driven ion doping behavior within the P3HT channel layer. Figure 2c illustrates the spatiotemporal evolution of the doping process across four distinct stages. Under a no‐bias condition (0 V), the ion gel remains in electrical equilibrium, with [TFSI]^−^ anions randomly dispersed and immobile (stage i). With a voltage of −0.5 V applied to the drain terminal, [TFSI]^−^ anions migrate toward the source electrode, leading to initial anion accumulation and partial localized doping (stage ii). When a gate voltage of −2 V is subsequently applied, a massive number of anions migrate into the P3HT channel, inducing progressive electrochemical doping and continuous redox reactions. This process dynamically regulates the optical absorption of the film and triggers a visible electrochromic alteration propagating across the channel (stage iii). Following complete doping, the electrochemical reactions stabilize, and the channel layer fully transitions from its initial purple state to a highly transparent, colorless state (stage iv).

As shown in Figure 2d, the normalized length of the doped region increases nearly linearly over time during stages ii and iii. This linear progression indicates a constant front velocity (v), which is governed by the product of effective ionic mobility (µ), and local lateral electric field (E) following the relation *v = µ · E* [38, 39]. These observations confirm that lateral ion migration is the rate‐limiting step for device turn‐on, consistent with ion‐transport‐limited models. Meanwhile, channel conductance scales with the length of the doped segment, as electrical conduction is dominated by the doped region, yielding *G(t)∝ x(t)*. This relationship is further validated by the synchronized growth curves of conductance and normalized length in Figure 2d.

### 2.2 Bionic and Display Functions of Ionochromic Neuromorphic Transistor

**FIGURE 3:**
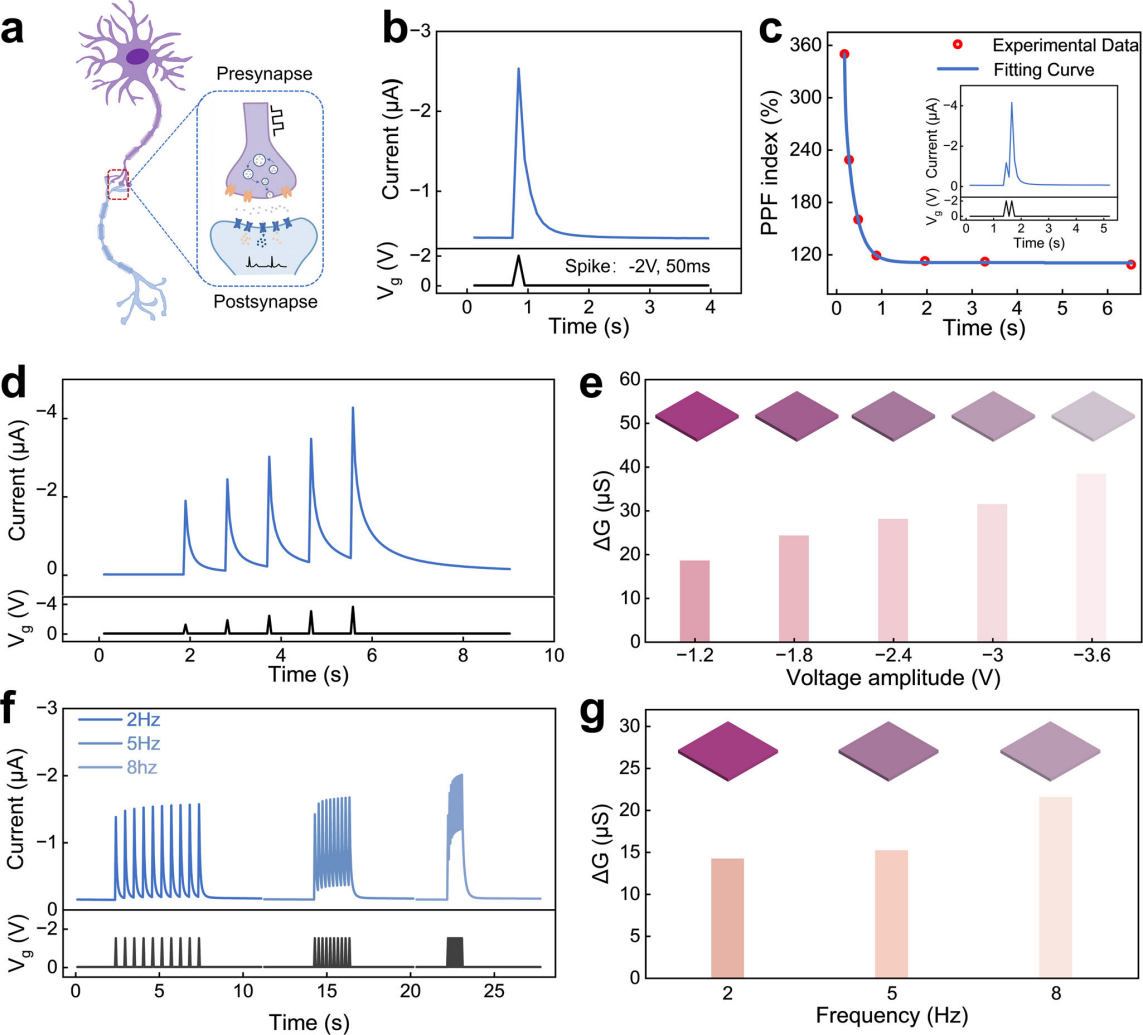
Synaptic functions and ionochromic properties of the neuromorphic transistor. (a) Schematic diagram of a biological synapse. (b) The EPSC induced by an electrical pulse. (c) PPF index (*A_2_/A_1_*) as a function of the inter‐pulse interval. (d) EPSC responses under electrical pulses with different amplitudes ranging from −1.2 to −3.6 V. (e) Amplitude‐dependent conductance modulation (*ΔG*), and the corresponding ionochromic evolution. (f) EPSC responses to pulse trains with varying frequencies. (g) Frequency‐dependent conductance changes (*ΔG*), and the corresponding ionochromic evolution.

To explore the biomimetic potential of the ion‐gated transistor, a series of electrical pulse stimulation protocols was designed to evaluate the performance of the ionochromic neuromorphic transistor. In biological synapses, signal transmission relies on ion transport, where presynaptic electrical stimuli are processed within the synaptic cleft to generate excitatory postsynaptic currents (EPSCs) that underpin synaptic plasticity (Figure 3a). Analogously, [TFSI]^−^ anions in the ion‐gel gate dielectric are driven into the channel under a gate electric field, modulating channel conductance and enabling artificial emulation of synaptic plasticity. Figure 3b shows the response of the device to a single presynaptic pulse (V_p_ = −2 V, t_p_ = 50 ms). The stimulus triggers an instantaneous increase in EPSC from −0.1 to −2.5 µA, followed by relaxation to the baseline within ∼1.5 s which effectively replicates the short‐term plasticity (STP) observed in biological synapses.

Short‐term memory (STM) characteristics were further examined via a paired‐pulse facilitation (PPF) protocol. As shown in the inset of Figure 3c, the peak current elicited by the second pulse (*A_2_*) significantly exceeds that of the first pulse (*A_1_*). This facilitation arises from the relatively slow ion dynamics in the electrolyte, where shorter inter‐pulse intervals lead to residual ion accumulation that enhances the subsequent response. The temporal dependence of the PPF effect was quantified by fitting the *A_2_/A_1_* index decay as a function of inter‐pulse interval (*Δt*) with a double‐exponential function (Figure 3c), yielding relaxation time constants of τ_1_ = 0.016 s and τ_2_ = 0.23 s. Beyond the transient single‐pulse and paired‐pulse STP responses, the device also exhibits controllable multi‐level conductance programming under consecutive gate pulses (V_G_ = −3.0 V, 50 ms). The conductance states were extracted after the fast transient relaxation reached a relatively stable plateau. The programmed conductance states remain distinguishable over 100 s with a maximum relaxation of only ∼3.52% (Figure S10), demonstrating measurable short‐to‐intermediate‐term weight retention for temporary dynamic visual‐memory applications.

Moreover, the dynamic information processing capabilities and corresponding real‐time ionochromic behavior were evaluated by examining the responses of the device under different electrical stimulus conditions. Amplitude‐dependent plasticity is critical for weighted signal encoding and analog information processing in neuromorphic systems. Accordingly, single voltage pulses with amplitudes ranging from −1.2 to −3.6 V were applied (Figure 3d), and results showed that peak EPSC increases monotonically with stimulus amplitude. Quantitative analysis (Figure 3e), where conductance change (*ΔG*) was extracted from Figure 3d, revealed a stepwise enhancement in *ΔG* from 18.6 to 38.4 µS. Notably, each conductance state correlates with a distinct optical state. The inset illustrates five distinct color states (*I*–*V*) recorded after applying different stimulus amplitudes, confirming a direct mapping between conductance weight (*ΔG*) and visual output.

The temporal sequence processing capability of the device was examined using pulse trains with frequencies of 2, 5, and 8 Hz (Figure 3f). The device exhibited frequency‐dependent potentiation. For instance, at 2 Hz, minimal conductance variation (*ΔG* = 14.5 µS) and slight ionochromic behaviors were observed (inset of Figure 3g). This is attributed to limited ionic accumulation due to sufficient relaxation between successive pulses. At 8 Hz (higher frequency), conductance change reached a maximum (*ΔG* = 21.6 µS) with the most pronounced color shift, achieving an approximately 1.5‐fold modulation in conductance compared to the 2 Hz stimulation. This behavior indicates that ionic doping has approached saturation. These findings prove that pulse amplitude and frequency effectively regulate ion accumulation in the channel. Higher amplitudes and frequencies induce greater ionic doping, directly determining the magnitude of conductance modulation and the depth of ionochromic transition.

To systematically evaluate the overall performance of the ionochromic neuromorphic transistor, key electrical, and electrochromic indicators were further quantified, including response time, cycling stability, event energy, optical contrast, and coloration efficiency (Figure S11). These results demonstrate a balanced performance in response speed, cycling stability, energy consumption, and optical modulation, supporting the potential of the device for visually interactive neuromorphic applications.

Benefiting from the excellent uniformity of the synaptic transistors, a 4 × 4 ionochromic neuromorphic transistors array was fabricated (Figure S12a). The electrical uniformity of the array was evaluated by characterizing 32 randomly selected devices from the same batch (Figure S12b), which exhibited highly consistent transfer characteristics, confirming reliable device stability. From the transfer curves, threshold voltage (*V_th_*), subthreshold swing (*SS*), and on/off ratio were extracted and summarized in Figure S13a–c; their statistical distributions provide a crucial metric for evaluating electrical uniformity (calculation formulas are provided in Note S1 [40, 41]). The average values for these 32 devices are: *I_on_/I_off_* = (1.7 ± 0.3) × 10^3^, *SS* = 509 ± 50 mV dec^−^^1^, and *V_th_* = −(0.7 ± 0.1) V. These results suggest a highly uniform device, whose response characteristics enable accurate information encoding and reliable visualization for interactive applications.

### 2.3 On‐Site Perception and Feedback for Morse Code Signals

**FIGURE 4:**
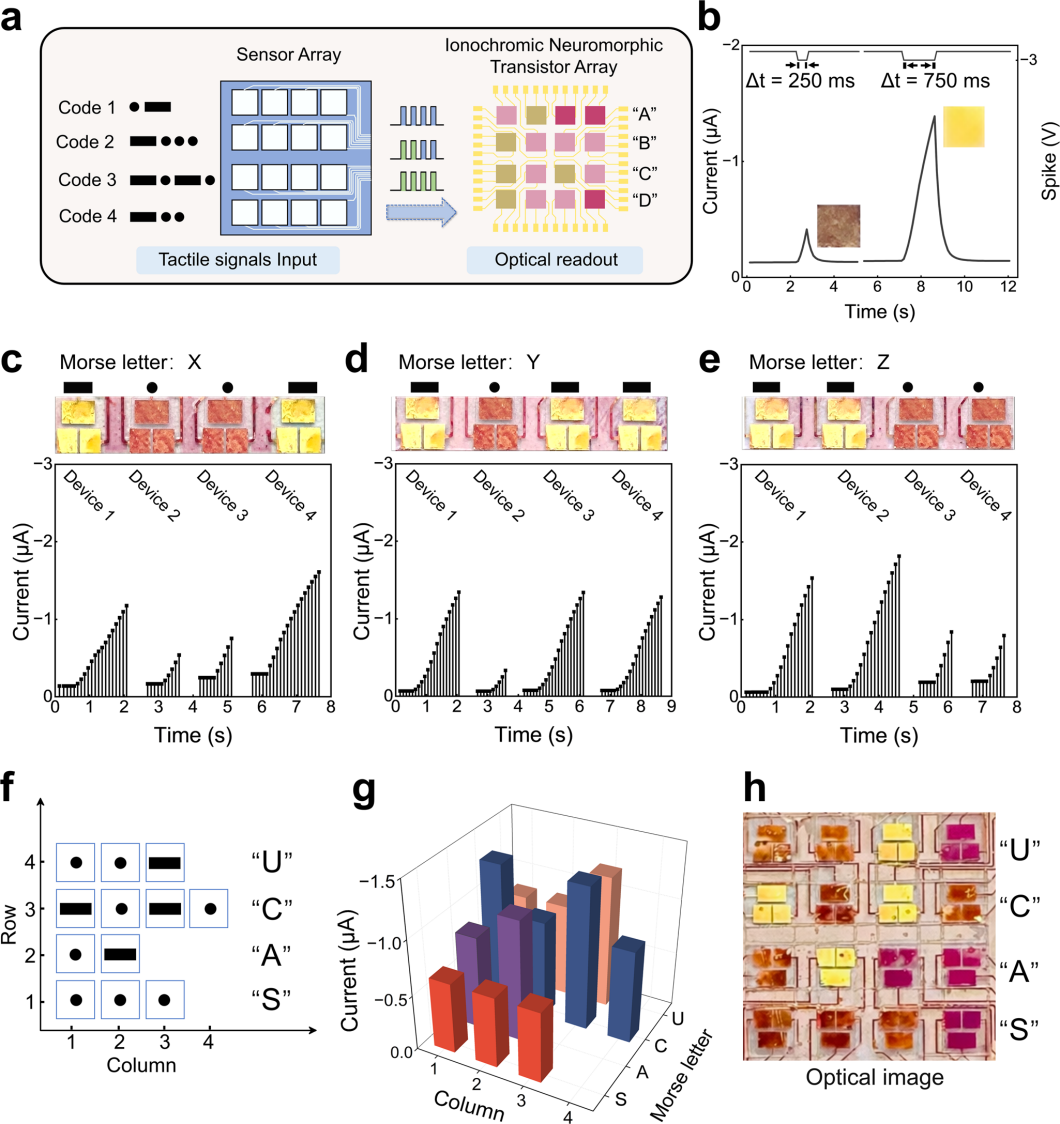
On‐site demonstration of perception and feedback for Morse code signals. (a) Schematic illustration of the Morse code tactile–visual conversion system, demonstrating the transformation of Morse‐encoded signals into distinctly ionochromic patterns. (b) EPSCs triggered by two spikes with durations of 250 and 750 ms. (c–e) EPSC responses of the ionochromic neuromorphic transistor array to pulse sequences encoding the Morse letters "X", "Y", and "Z", respectively. The top insets display the visualization of encoded information through corresponding ionochromic patterns. (f) Pulse sequences used to encode the word "UCAS". (g) EPSC outputs of the array triggered by sequential pulse trains representing the letters "U", "C", "A", and "S". (h) Optical photograph of the 4 × 4 array after programming with the "UCAS" sequence, where the encoded information is directly visualized through the resulting ionochromic patterns.

The ionochromic neuromorphic transistor establishes a new paradigm for on‐site visualization, directly transducing electrical signals into dynamic color changes and enabling intuitive interpretation via spatial ionochromic patterns. To enhance the visual readout and clarity of electrochromic behavior, especially for the P3HT film under gradually increased transparency, a thin, patterned Au film with a bright yellow hue was integrated into the backside of the device as a high‐contrast background. This design facilitates reliable discrimination between distinct electrochromic states. Subsequently, a visual Morse code system was developed (Figure 4a), where ‘dot’ and ‘dash’ symbols were emulated by electrical pulses of specific durations (250 ms for dots, 750 ms for dashes) at a fixed amplitude of −1.5 V. This pulse‐width‐dependent stimulation modulates the extent of ionic doping in the channel, generating readily distinguishable ionochromic responses across the transistor array. Figure 4b presents the EPSC responses and corresponding color states induced by two presynaptic spikes, specifically a 250 ms pulse (dot) and a 750 ms pulse (dash). The longer dash pulse triggers a substantially higher EPSC peak and induces a pronounced ionochromic change in the P3HT film, whereas the dot pulse produces only a subtle discoloration. This stark optical contrast enables clear visual differentiation between the two fundamental Morse code elements.

Leveraging this encoding rule, complex characters were successfully represented via specific pulse sequences. Figure 4c–e illustrates the electrical encoding and optical visualization of the letters “X”, “Y”, and “Z”. For each character, the EPSC peak currents confirm the successful encoding of the corresponding Morse code sequences, while the ionochromic patterns of the device permit intuitive character recognition. Specifically, dash pulses induce pronounced discoloration, and dot pulses generate weaker changes, ensuring each character is uniquely represented by a distinct ionochromic mode.

To further demonstrate the multi‐letter encoding capability of the visual typewriter, the word “UCAS” was programmed across the 4 × 4 transistor array. Figure 4f displays the input pulse sequences used to encode the four letters, with each row assigned to one character. Multi‐channel EPSC recordings during programming confirmed reliable and independent addressing of all devices (Figure 4g). The optical image of the array (Figure 4h) reveals unique ionochromic modes for each letter, enabling straightforward recognition of the encoded word. Collectively, these results validate that the ionochromic neuromorphic transistor array supports temporal pulse‐encoded optical responses and parallel optical visualization. This capability allows information encoded via pulse sequences to be intuitively retrieved from spatial color patterns, laying a foundation for next‐generation intelligent perception systems that integrate neuromorphic computing with visual feedback.

### 2.4 Intelligent Robotic Arm Recognition Integrating Ionochromic Neuromorphic Array

**FIGURE 5:**
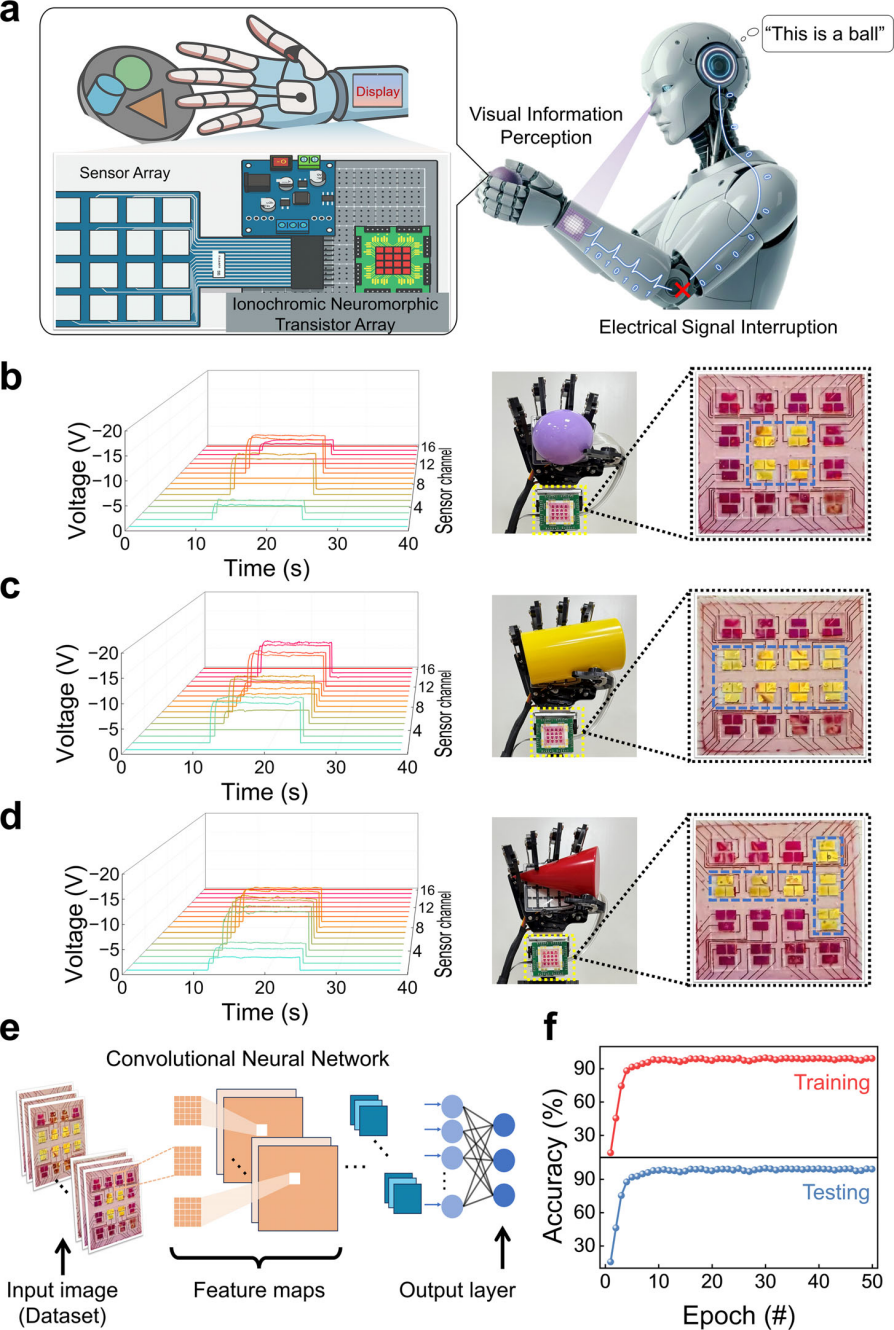
High‐precision intelligent robotic arm recognition system integrating ionochromic neuromorphic array. (a) Illustration of tactile signal visualization, where pressure signals from an object are transduced into spatially ionochromic patterns by the ionochromic neuromorphic transistor array for object recognition. (b–d) Experimental demonstrations of object recognition based on an integrated robotic hand using (b) a sphere, (c) a cylinder, and (d) a cone. For each object, the description includes the real‐time multichannel voltage signals recorded from the pressure sensor array on the left, whereas the corresponding photograph of the grasping process and the ionochromic pattern are displayed on the right. The latter is highlighted by blue dashed boxes. (e) Schematic architecture of the CNN employed for object classification. (f) Classification accuracy as a function of training iterations during network training and testing.

Current state‐of‐the‐art tactile perception systems predominantly rely on continuous multi‐channel electrical sampling, complex back‐end signal conditioning, and digital post‐processing to extract and recognize object features, which introduces significant energy consumption, signal latency, and system complexity [42]. In stark contrast, the ionochromic neuromorphic transistor array allows direct mapping of spatial pressure variations into discriminative color responses, realizing intelligent tactile visualization with in situ spatial feature extraction, representing a paradigm shift in tactile perception architecture. To validate the applicability of the ionochromic neuromorphic transistor array for tactile perception, we integrated it with a flexible tactile sensing platform (Figure 5a), forming a closed‐loop tactile visualization system. The operational mechanism relies on a series integration of the resistive tactile sensor and the synaptic transistor. When mechanical pressure is applied, the resistance of the sensor drops, which increases the voltage applied to the gate of the transistor, triggering the ion transport process and the corresponding localized color transition.

The perception feedback performance of the system was evaluated using objects with distinct geometric morphologies (sphere, cylinder, and cone; Figure 5b–d), as their structures induce various spatial pressure distributions upon contact, critical for differentiating object features. When the robotic hand grasps these objects, the tactile sensor array converts the contact pressure into localized electrical signals, which are directly transduced by the ionochromic neuromorphic transistor array into intuitive optical patterns. As a result, the array can be functioned as a high‐efficiency spatial‐to‐visual encoder, translating pressure profiles into distinguishable 2D color signatures without the need for complex back‐end computations. Specifically, grasping a symmetric sphere concentrates contact force at the center of the array, as the spherical geometry distributes pressure radially. This centralized pressure signature is visually reconstructed as a distinct local 2 × 2 ionochromic pattern (Figure 5b, right), which directly reflects the pressure concentration. More importantly, this localized response precisely validates the high spatial resolution and in situ feature extraction capability of the device array. It thereby proves the system as an efficient spatial‐to‐visual encoder, mapping 3D geometric profiles directly to 2D color signatures while circumventing complex backend computations. In contrast, a cylindrical object induces an elongated contact area along its longitudinal axis, as the cylindrical surface maintains line contact with the array. The transistor array captures this spatial feature as a continuous 2 × 4 rectangular ionochromic pattern spanning adjacent rows (Figure 5c, right). Similarly, grasping a cone generates a spatially asymmetric pressure distribution that tapers from the tip (high pressure) to the base (low pressure). The array inherently captures this structural asymmetry, producing a distinct T‐shaped ionochromic profile (Figure 5d) that encodes the tapering geometry of the cone. Notably, the integrated pressure inputs accumulate over time due to the synaptic STP characteristics of the device, amplifying the color contrast and reinforcing the distinctiveness of the spatial patterns. The corresponding multi‐channel electrical signals recorded during these grasping processes, which validate the pressure‐to‐optical mapping, are provided in Figure 5b–d and Figure S17.

To quantitatively validate the reliability of object recognition based on these ionochromic patterns, the generated optical images were employed as input database for a custom designed convolutional neural network (CNN) (Figure 5e). The network processes 64 × 64 RGB images through two sequential convolutional layers utilizing 32 and 64 filters of 3 × 3 size, respectively. Each convolutional step is followed by a 2 × 2 max pooling layer for spatial downsampling. The extracted features are then flattened and passed into a fully connected layer of 128 neurons, culminating in an output layer of 3 neurons to classify the specific object geometries. The classification accuracy for object shape recognition was monitored as a function of training iterations (Figure 5f), revealing a rapid and monotonic increase in accuracy, which plateaued at 98.4% after 40 iterations. This high classification accuracy confirms that the in situ ionochromic display effectively encodes tactile information into visually discriminative features, enabling robust object recognition without reliance on centralized data processing or electronic signal transmission.

The proposed ionochromic neuromorphic transistor integrates synaptic information processing with direct visual output, offering functional integration beyond conventional ion‐modulated devices. This work combines tactile sensing, synaptic response, and optical visualization into a single hardware platform, eliminating the traditional separation between sensor, processor, and display. By merging ionic transport‐driven synaptic plasticity with electrochromic optical readout, the system performs analog in‐memory computing directly at the sensor level, where pressure information is immediately translated into a perceivable visual format. This architecture is particularly advantageous for resource‐constrained edge devices and real‐time tactile perception tasks (e.g., robotic manipulation, wearable electronics), where speed, power efficiency, and intuitive feedback are paramount. Nevertheless, further optimization is still needed. In the current system, ion migration in the ion‐gel electrolyte remains relatively random, which may cause non‐uniform doping dynamics in the P3HT channel. In addition, the channel/electrolyte interface and device layout should be further optimized to improve signal consistency. Future work will focus on tailoring the ion‐gel composition, optimizing the channel interface and device architecture, and developing robust encapsulation methods to improve long‐term reliability and enable larger‐scale tactile‐visual conversion arrays.

## 3 Conclusion

In summary, we report an ionochromic neuromorphic transistor enabling integrated tactile‐to‐visual information conversion. By leveraging voltage‐driven [TFSI]^−^ ion doping in electrochromic P3HT channels, the device shows synchronous modulation of electrical conductance and optical coloration. Morse‐coded pulse sequences applied to the transistor array can be translated into discriminative ionochromic patterns, enabling intuitive optical readout without complex decoding. When integrated into a robotic tactile perception system, the array faithfully converts grasping‐induced spatial pressure distributions into geometry‐specific ionochromic signatures, which achieves a 98.4% object recognition accuracy via neural network validation. This work establishes a viable strategy for integrating tactile sensing and optical visualization within compact neuromorphic electronics, providing a promising closed‐loop technological pathway toward advanced embodied intelligence and intelligent perception systems.

## 4 Methods

### 4.1 Materials

Poly (vinylidene fluoride)‐hexafluoropropylene (P(VDF‐HFP)) was purchased from Macklin. 1‐Ethyl‐3‐methylimidazolium bis (trifluoromethylsulfonyl) imide ([EMIM][TFSI]) was obtained from Energy Chemical. Poly(3‐hexylthiophene‐2,5‐diyl) (sterically regulated) (P3HT) and dichlorobenzene (98%) were purchased from Aladdin. Acetone was obtained from Sinopharm Chemical Reagent Co., Ltd. All chemicals and solvents were used as received without any further purification.

### 4.2 Preparation of Ion‐Gel Polymer Electrolytes

PVDF‐HFP was dissolved in acetone and stirred at 50°C for 4 h. The ionic liquid [EMIM][TFSI] was then added to above mixture to obtain a composite solution with a weight ratio of 1:4, followed by continued stirring at 50°C for another 4 h. The mixture was spin‐coated onto a clean glass substrate and annealed at 80°C overnight to produce a flexible ion‐gel electrolyte film. Before device fabrication and testing, all ion‐gel films were dried in a vacuum oven at 70°C for 24 h to reduce the influence of residual water and improve the reproducibility of the ionic response. The average thickness of the spin‐coated ion‐gel film was approximately 25 µm.

### 4.3 Preparation of P3HT Solution

P3HT was dissolved in a mixed solution of dichlorobenzene and chloroform at a concentration of 10 mg/ml and stirred at 50°C for 4 h.

### 4.4 Fabrication of Devices

The individual device was fabricated on cleaned PET substrates. First, 60 nm‐thick Au source, drain, and gate electrodes were deposited via thermal evaporation using a shadow mask. The channel length and width of the individual transistor were 100 and 1000 µm, respectively. Before P3HT spin coating, oxygen plasma treatment was used as a surface‐cleaning step to remove possible surface contaminants and improve the film‐forming quality of the subsequently spin‐coated P3HT layer. The oxygen plasma treatment was performed at 150 W for 5 min, with an O_2_ flow rate of 150 sccm and a chamber pressure of 470 mTorr. After plasma treatment, the substrates were immediately used for P3HT spin coating to reduce possible surface recontamination. The P3HT solution was spin‐coated at 2000 rpm for 20 s and then annealed at 70°C for 2 h. Finally, an ion‐gel was transferred to cover the channel and gate electrodes.

For the transistor array, the electrode patterns were defined via standard photolithography, followed by the deposition of 60 nm‐thick Au source, drain, and gate electrodes using thermal evaporation. The integrated array was designed as a 4 × 4 matrix, and each pixel unit adopted the same channel geometry as the individual device. To enhance optical contrast for visual observation of P3HT ionochromic properties, a patterned Au film was embedded on the backside of the array as a background. Other fabrication steps for the array remained consistent with the individual device.

### 4.5 Measurements

All electrical characteristics and pulse tests were measured on a Lake Shore probe station using a Keithley 4200 semiconductor parameter analyzer under controlled ambient conditions. The laboratory temperature was maintained at approximately 20°C, and the relative humidity (RH) was controlled within 40%–60%. All optical imaging was performed using a CX40M. UV–vis absorption spectra were tested using a Lambda 950 spectrophotometer. Raman spectra were acquired using a Renishaw inVia Reflex at an excitation wavelength of 532 nm. The thickness and surface roughness of the ion‐gel films were characterized to provide complete structural parameters for device reproducibility.

## Conflicts of Interest

There are no conflicts to declare.

## Supporting information




**Supporting File**: advs76683‐sup‐0001‐SuppMat.docx.

## Data Availability

The data that support the findings of this study are available from the corresponding author upon reasonable request.
