# Supplementary material for: Bioinspired Ionochromic Neuromorphic Transistors for Robotic Intelligent Perception
Source: Adv Sci (Weinh). 2026 Jul 24:e76683. Online ahead of print. doi: 10.1002/advs.76683 (PMC13398131; doi:10.1002/advs.76683)
Supplement: Supplementary file 1 — Supporting File: advs76683‐sup‐0001‐SuppMat.docx. [file ADVS-9999-e76683-s001.docx]

Supporting Information

**Bioinspired Ionochromic Neuromorphic Transistors for Robotic Intelligent Perception**

*Quanxing Yao ^1,2,3^, Xiaojian Zhu ^1,2,3^*, Runsheng Gao ^1,2,3^* Qian Jiang ^1^, Cui Sun ^1^,* *Yi Du ^1^, Xuerong Liu ^1,2,3^, Lixun Wang ^4^, Haolong Li ^1^, Yuejun Zhang ^4^, and Run-Wei Li ^5^**

^1^ Zhejiang Key Laboratory of Magnetic Materials and Applications, Ningbo Institute of Materials Technology & Engineering, CAS, Ningbo 315201, China

^2^ Center of Materials Science and Optoelectronics Engineering, University of Chinese Academy of Sciences, Beijing 100049, China

^3^ University of Chinese Academy of Sciences, Beijing 100049, China

^4^ Faculty of Electrical Engineering and Computer Science, Ningbo University, Ningbo 315211, China

^5^ Eastern Institute of Technology, Ningbo 315200, China

E-mail: [zhuxj@nimte.ac.cn](mailto:zhuxj@nimte.ac.cn) , [rsgao@nimte.ac.cn](mailto:rsgao@nimte.ac.cn) , [rwli@eitech.edu.cn](mailto:rwli@eitech.edu.cn)


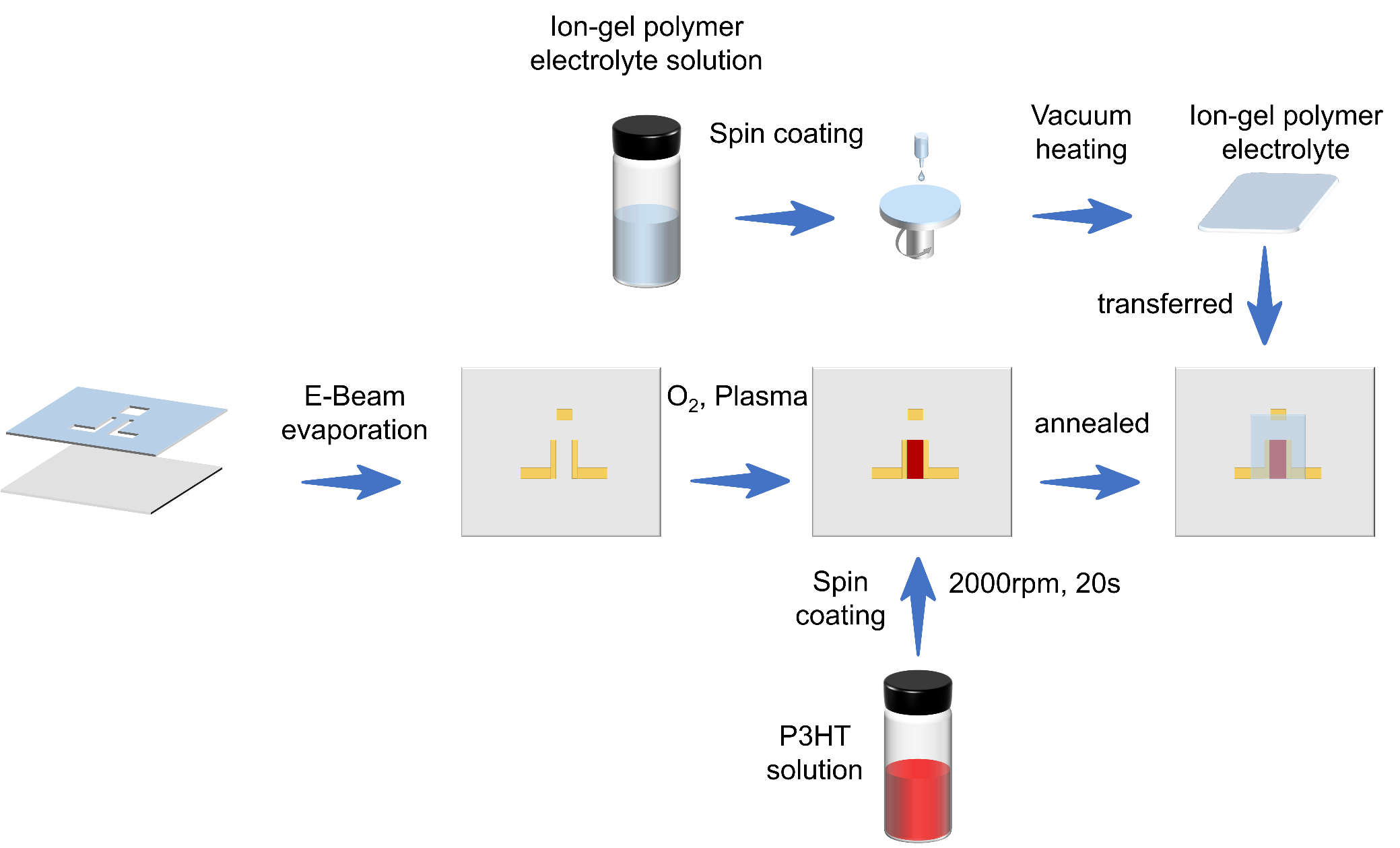


**Figure S1.** Fabrication process of the P3HT-based EGT device.


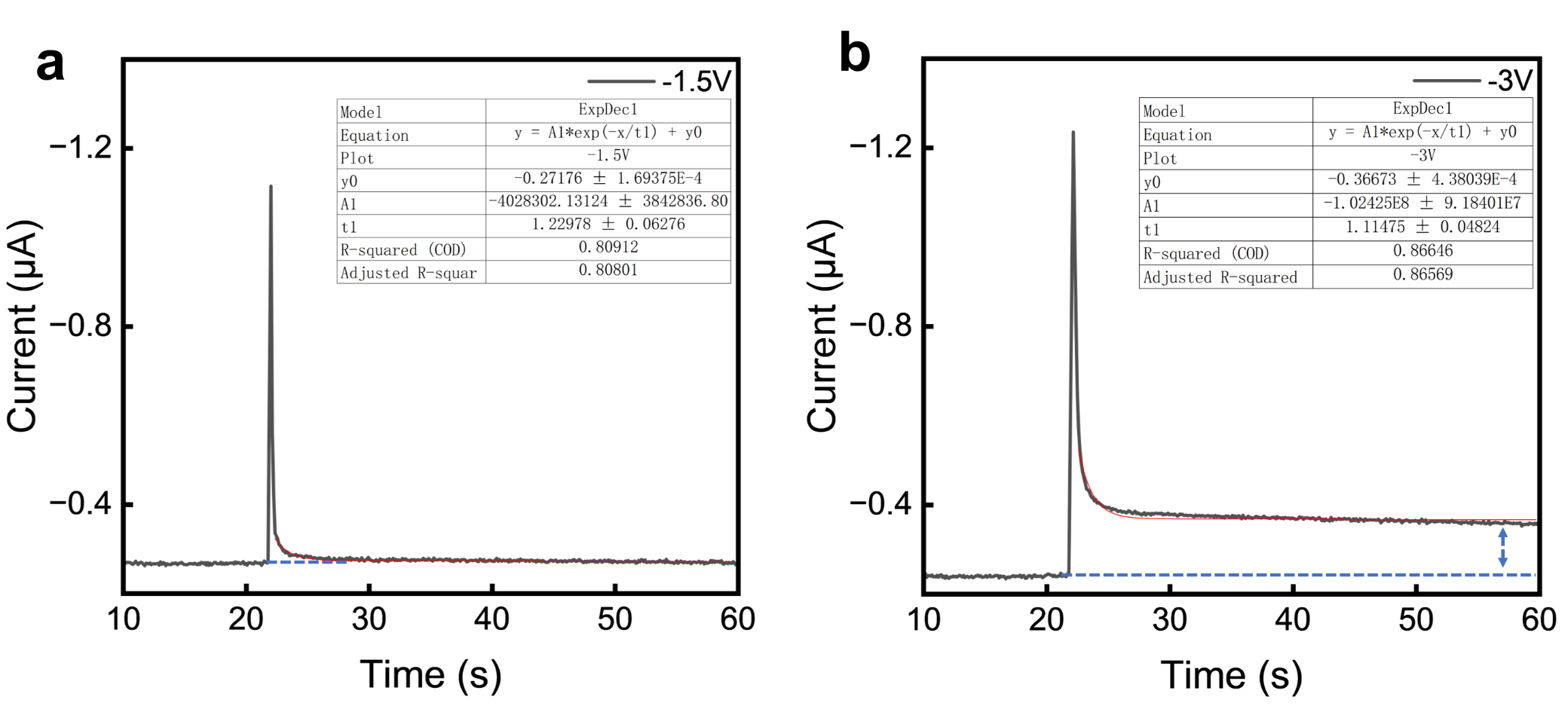


**Figure S2**. Transient current responses of the P3HT-based EGT device under gate pulses of (a) −1.5 V and (b) −3.0 V.


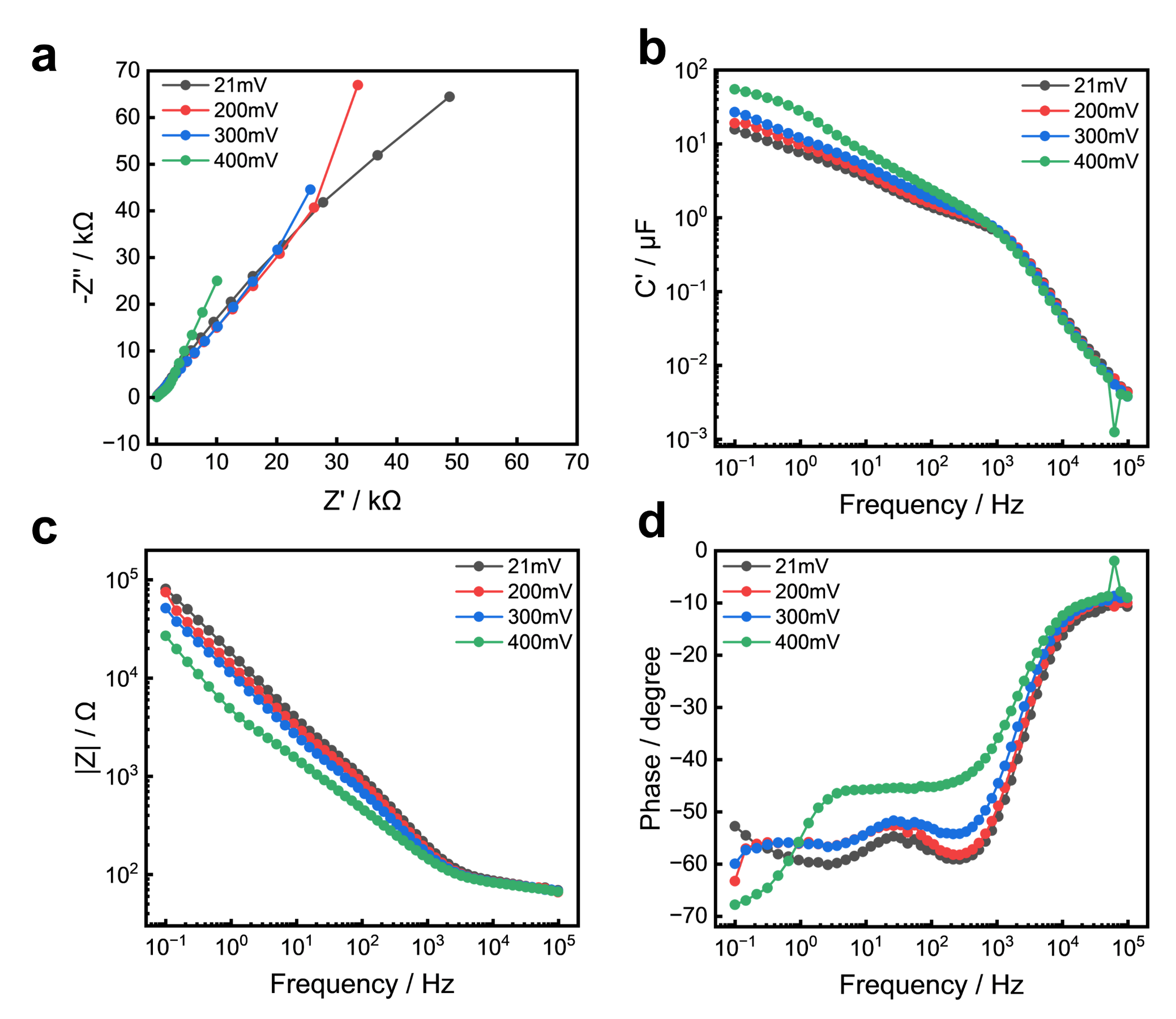


**Figure S3**. Electrochemical impedance spectroscopy characterization of the P3HT-based EGT device under different applied voltage amplitudes. (a) Nyquist plots, (b) frequency-dependent real capacitance C′, (c) impedance magnitude |Z|, and (d) phase angle measured under applied voltage amplitudes of 21, 200, 300, and 400 mV over the frequency range of 10⁻¹–10⁵ Hz.

Although the coupled conductance and optical changes demonstrate effective ionic modulation of the P3HT channel, they cannot fully identify the relative contributions of electrostatic double-layer modulation and electrochemical doping. Therefore, the device response was further analyzed by comparing pulse responses under different gate-voltage amplitudes and EIS results. As shown in **Figure S**2, the current response under −1.5 V rapidly decays back to the initial baseline after the removal of the gate pulse, with a relaxation time constant of 1.29978 s and a retained-current ratio of only 3.4 × 10⁻^3^. This indicates that low-voltage modulation is mainly governed by reversible electrostatic double-layer charging. In contrast, under −3.0 V stimulation, the current also shows a comparable fast relaxation time constant of 1.11475 s, confirming that double-layer relaxation still contributes to the initial decay process. However, the retained-current ratio increases to 0.204, indicating that a significant residual conductance state remains after removing the gate bias. This retained component is attributed to enhanced ion injection and electrochemical doping of the P3HT channel under high-bias stimulation. The EIS results further show pronounced frequency-dependent behavior, with low-frequency capacitance enhancement and diffusion-related impedance features (**Figure S**3), supporting the involvement of slow ionic redistribution under quasi-static conditions. These results indicate that low-voltage operation is mainly dominated by electrostatic double-layer coupling, whereas high-bias stimulation induces a mixed response in which electrochemical doping contributes to retained conductance modulation and synchronized electrochromic switching.


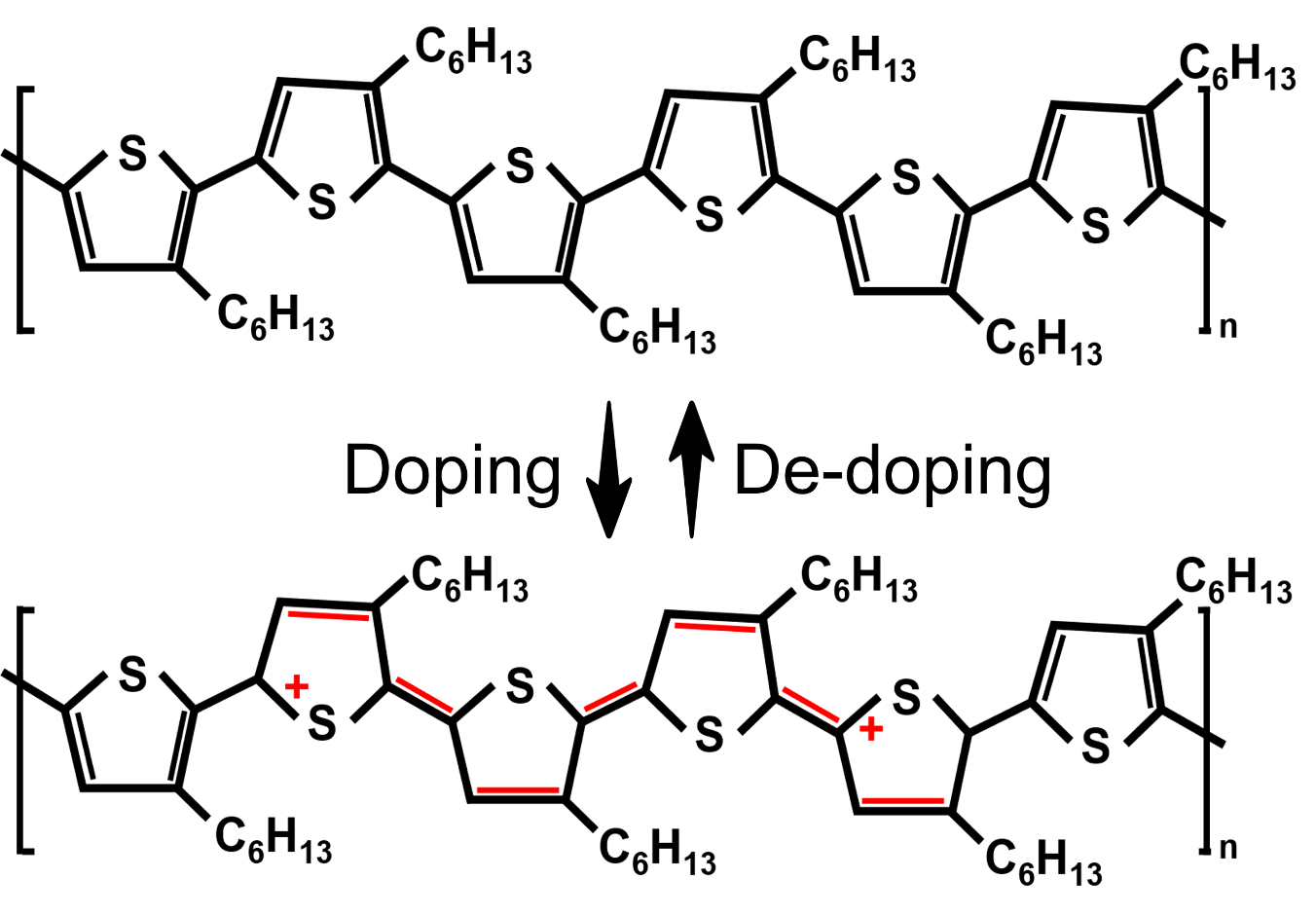


**Figure S4**. Schematic evolution of the P3HT band structure during the ion doping/de-doping process.


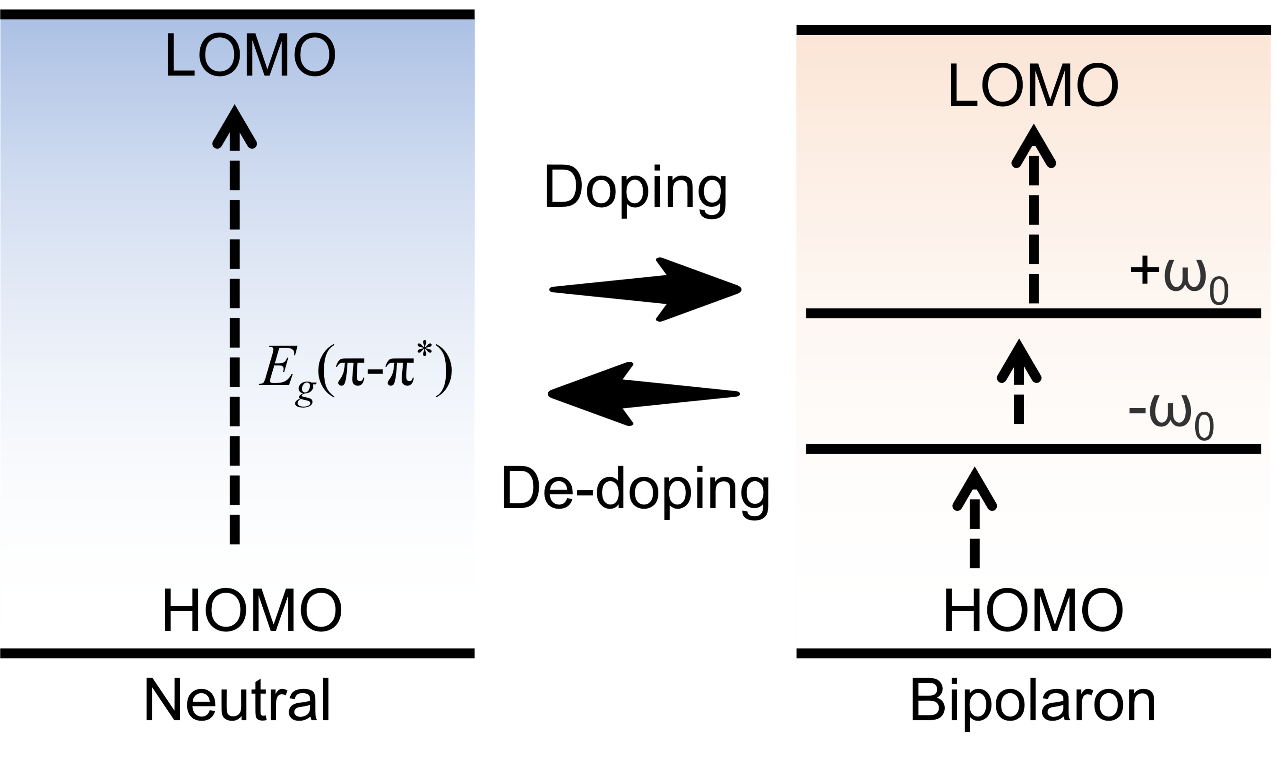


**Figure S5**. Reversible configuration transformation of P3HT induced by controlled ion doping.


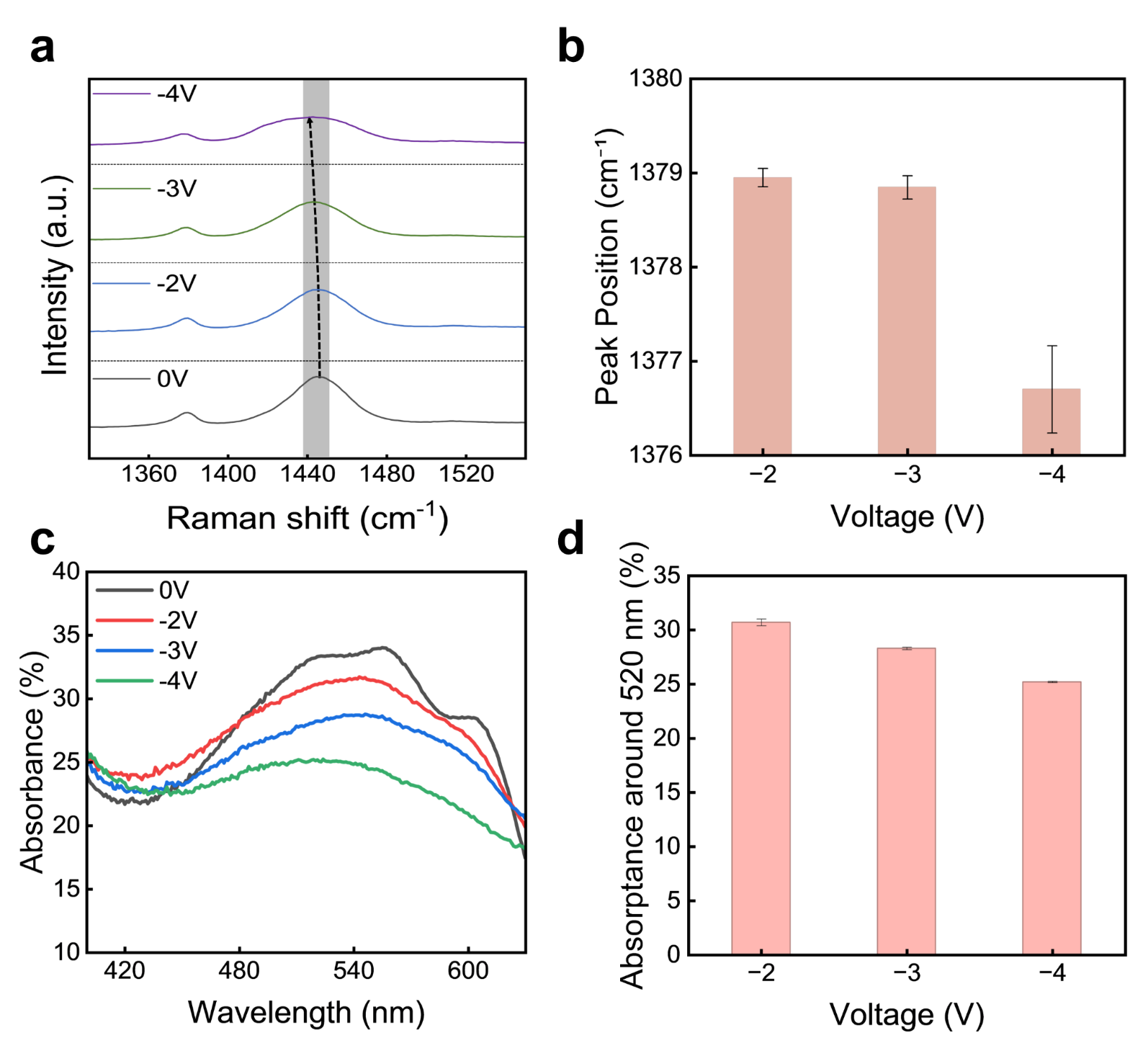


**Figure S6**. Raman and UV–vis characterization of P3HT under different gate voltages. (a) Raman spectra of P3HT recorded at 0, −2, −3, and −4 V. (b) Statistical analysis of the extracted Raman peak positions with error bars. (c) UV–vis absorption spectra of P3HT recorded at 0, −2, −3, and −4 V. (d) Statistical analysis of the absorbance around 520 nm under different gate voltages.


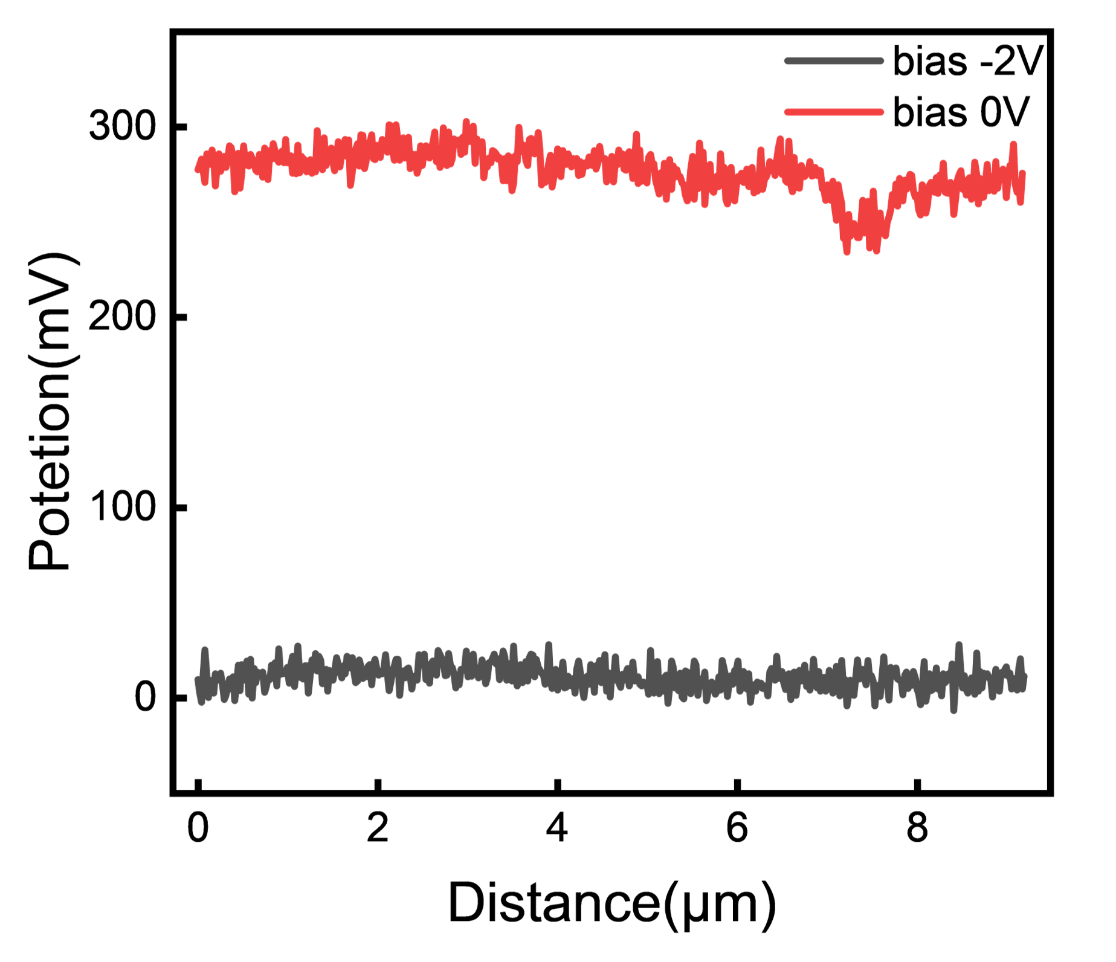


**Figure S7**. KPFM surface potential analysis of the P3HT film under different bias conditions. CPD line profiles extracted from the KPFM potential maps at 0 V and after applying a −2 V bias, showing a clear surface potential decrease under negative gate bias.


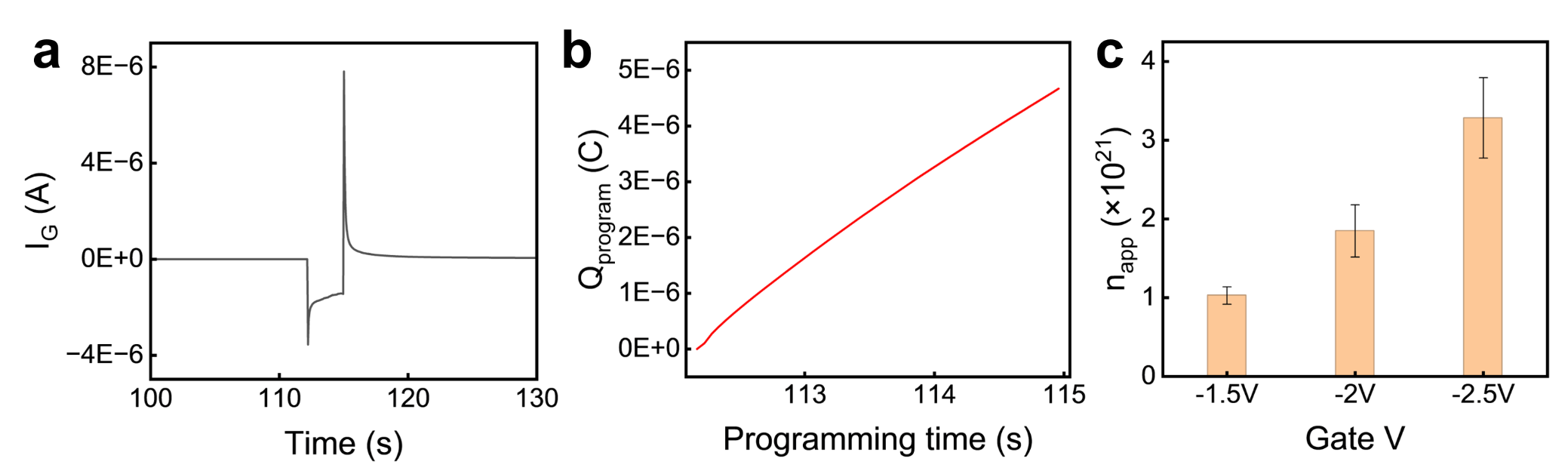


**Figure S8**. Quantitative estimation of the apparent doping concentration. (a) Gate-current response during the programming process. (b) Integrated programmed charge obtained from the time integration of the gate current. (c) Extracted apparent doping concentration under different gate voltages, with error bars.


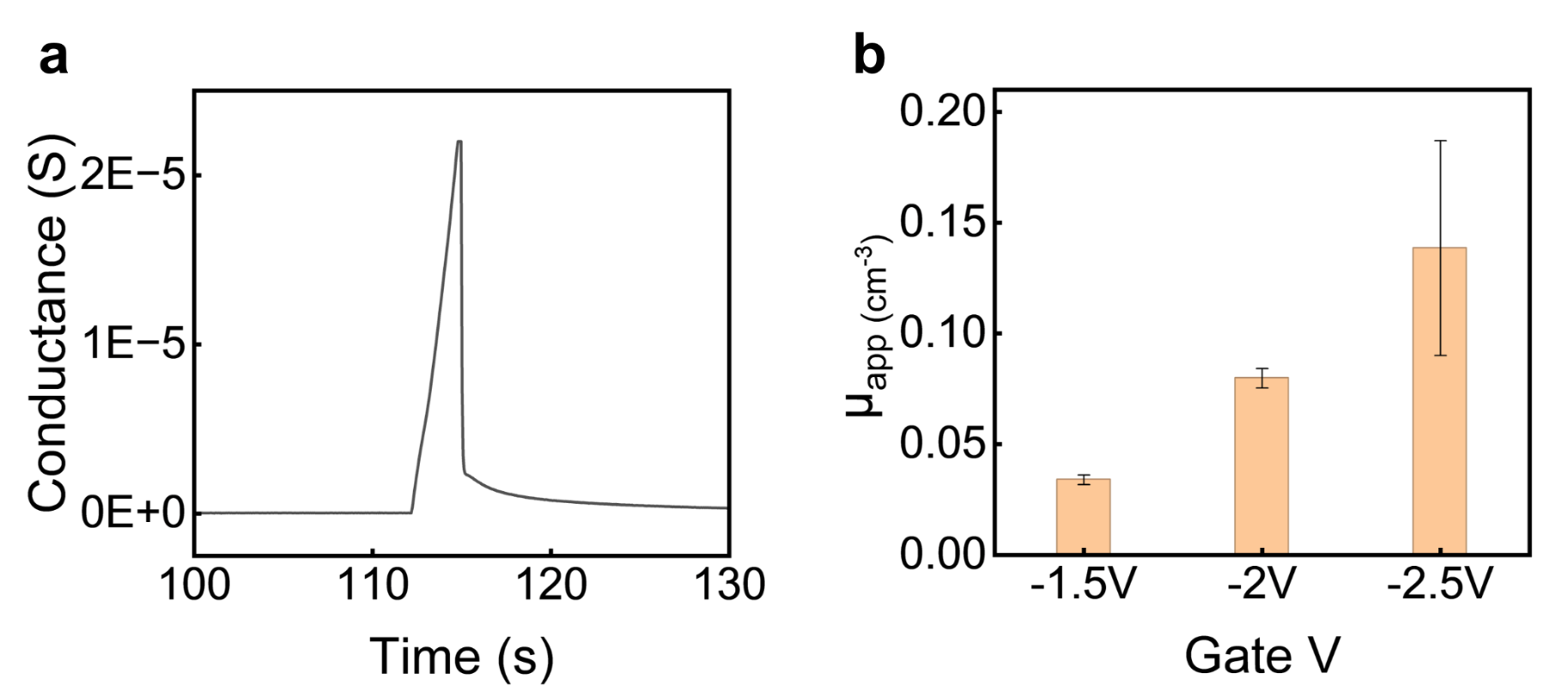


**Figure S9**. Estimation of apparent carrier mobility during electrochemical modulation. (a) Channel conductance variation during the programming process. (b) Calculated apparent carrier mobility under different gate voltages, with error bars.

To further quantify the gate-voltage-induced electrochemical modulation of the P3HT channel, Raman, UV–vis, apparent doping concentration, and apparent carrier mobility analyses were performed under different gate-voltage conditions. The Raman redshift, surface potential variation, and decreased visible absorption confirm the voltage-dependent modulation of the molecular and electronic states of P3HT (**Figure S**6 and **Figure S**7). Based on the programmed gate charge and channel conductance, the apparent doping concentration increases from approximately 1.0 × 10^21^ cm⁻^3^ at −1.5 V to 3.3 × 10^21^ cm⁻^3^ at −2.5 V, while the apparent mobility increases from approximately 0.035 to 0.14 cm^2^ V⁻^1^ s⁻^1^ (**Figure S**8 and **Figure S**9). These results quantitatively demonstrate that increasing the negative gate-voltage amplitude enhances the electrochemical doping level and effective carrier transport in the P3HT channel.


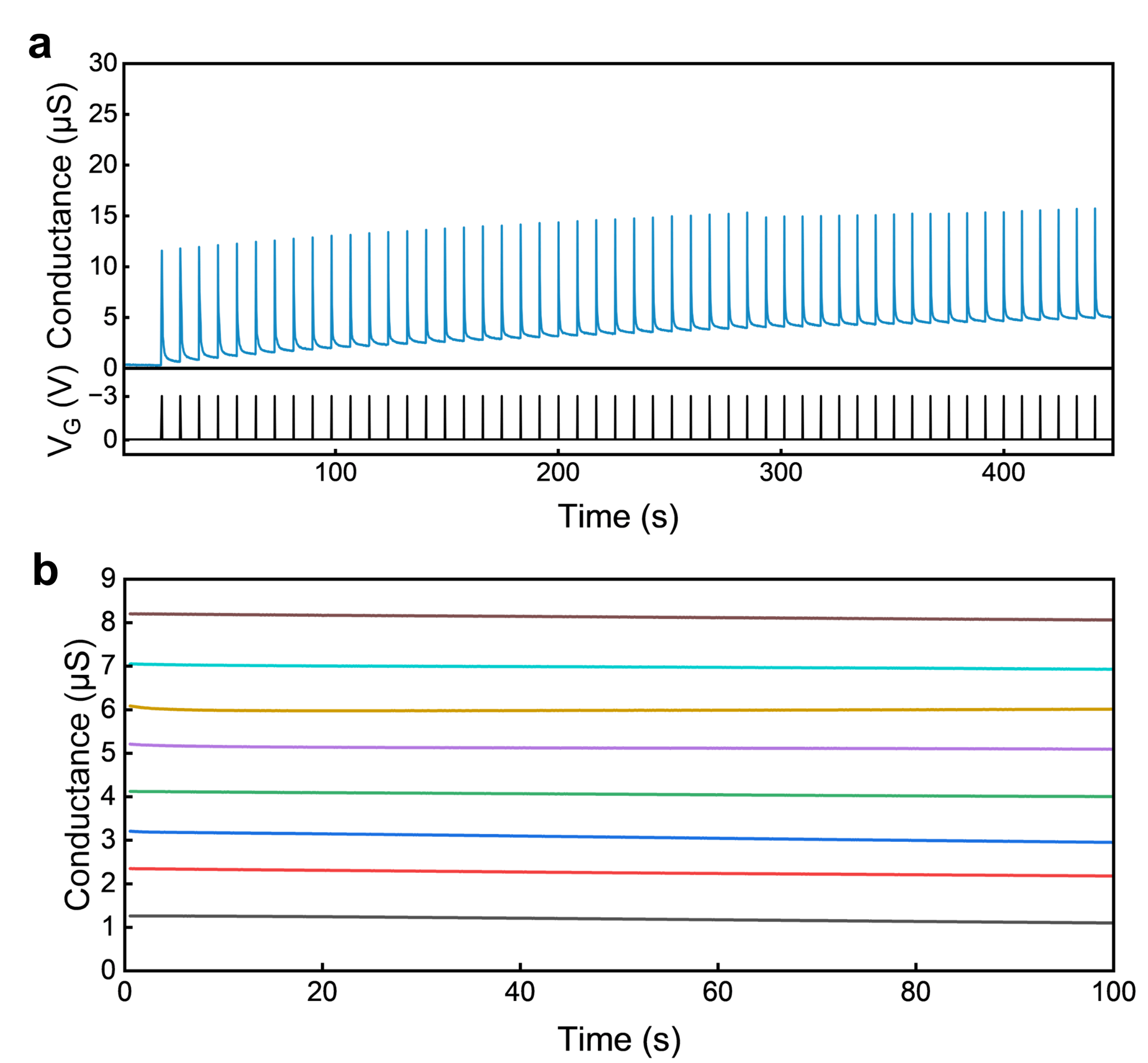


**Figure S10**. Multi-level conductance programming and retention characteristics of the P3HT-based EGT device. (a) Gradual conductance modulation under consecutive negative gate pulses (V_G_ = −3.0 V, 50 ms). (b) Retention behavior of different programmed conductance states measured over 100 s.


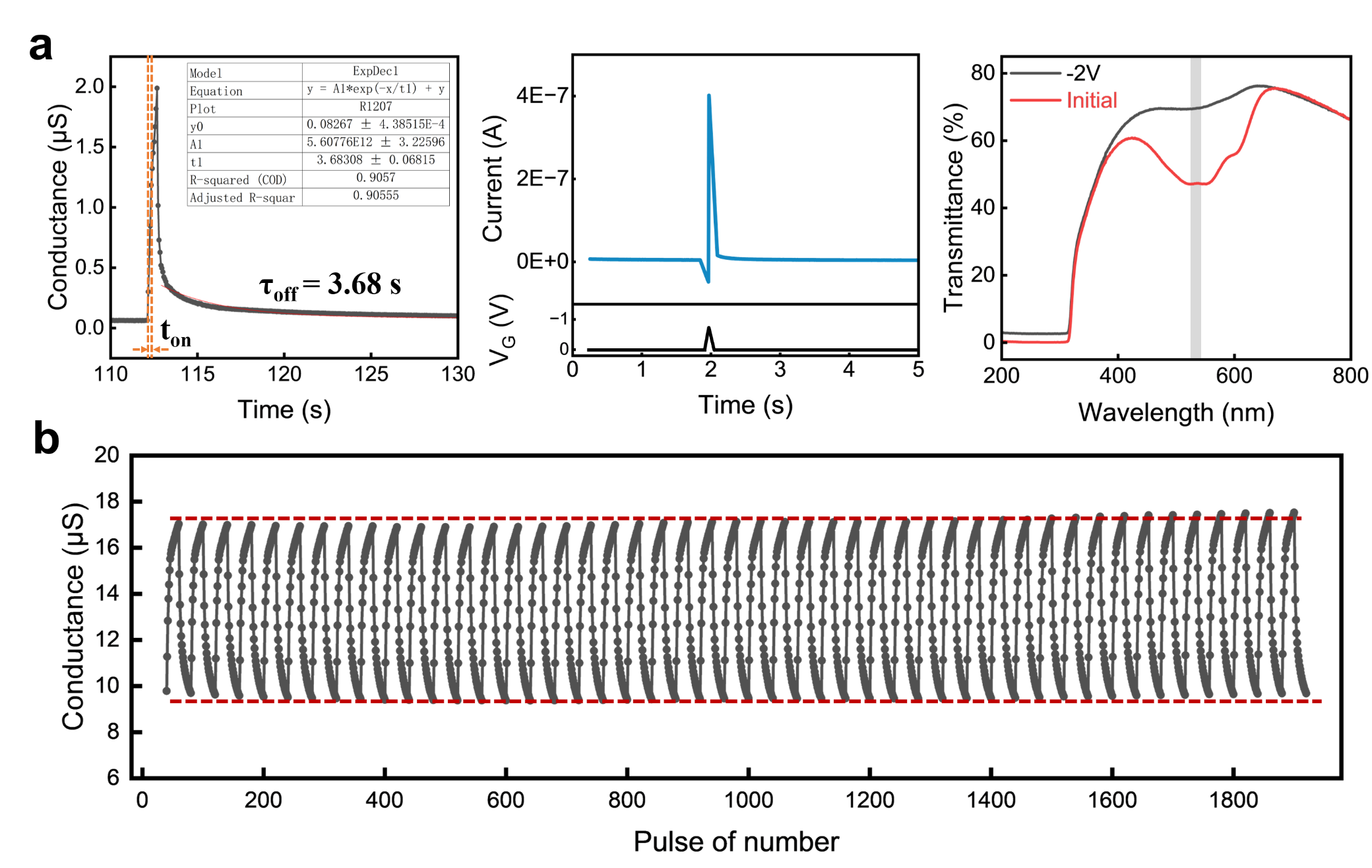


**Figure S11**. Performance evaluation of the P3HT-based EGT device. (a) Temporal conductance response and exponential relaxation fitting, showing a turn-on response time extracted from the 10%–90% conductance range and a relaxation time constant of τ_off_ = 3.68 s. (b) Cycling stability under repeated potentiation/depression pulses. (c) Transient gate-current response and gate-voltage waveform for event-energy estimation. (d) Optical transmittance spectra of the P3HT channel before and after applying V_G_ = −2 V, with the shaded region indicating the wavelength used for ΔOD and CE calculations.


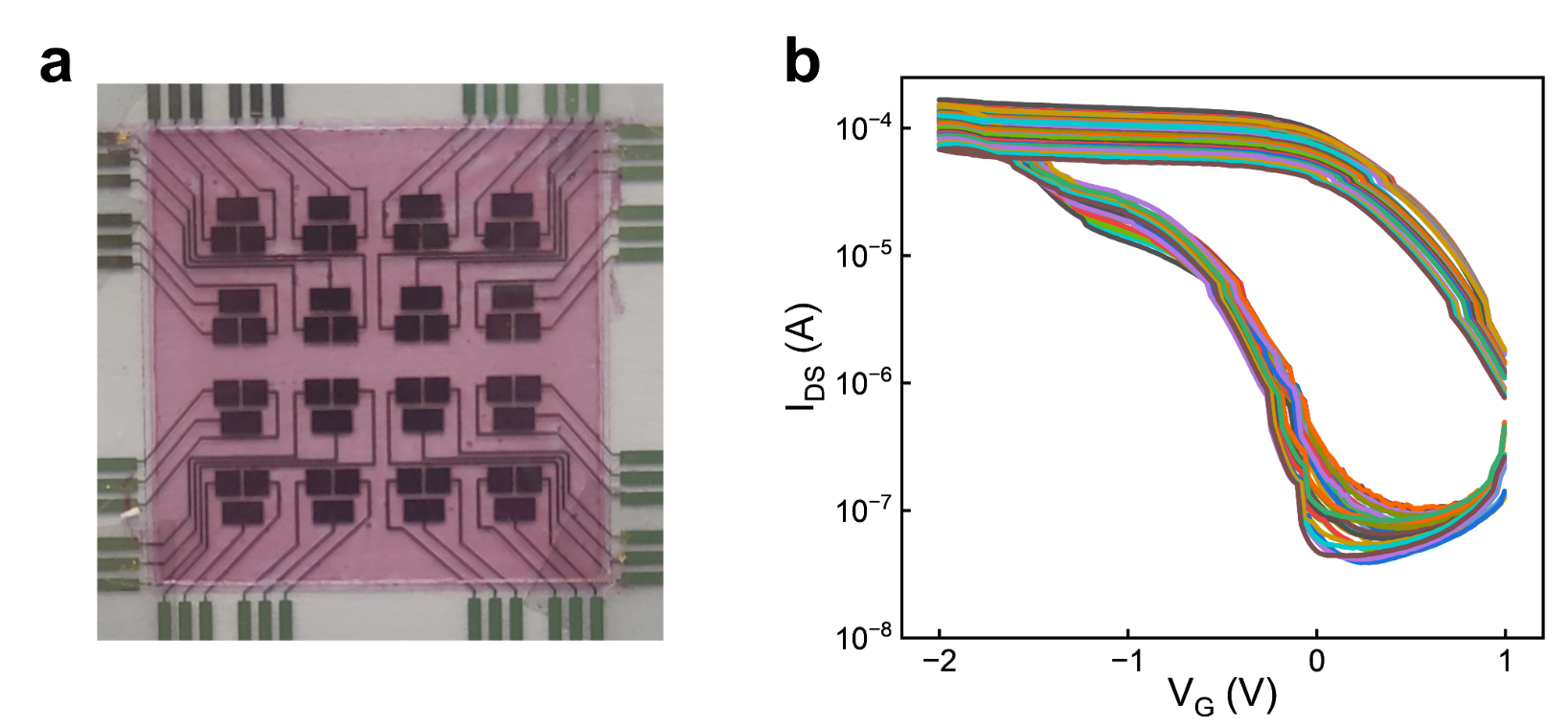


**Figure S12**. (a) Optical photograph of the 4×4 ionochromic neuromorphic transistor array. (b) Transfer characteristics comprising 32 curves obtained from randomly selected devices.


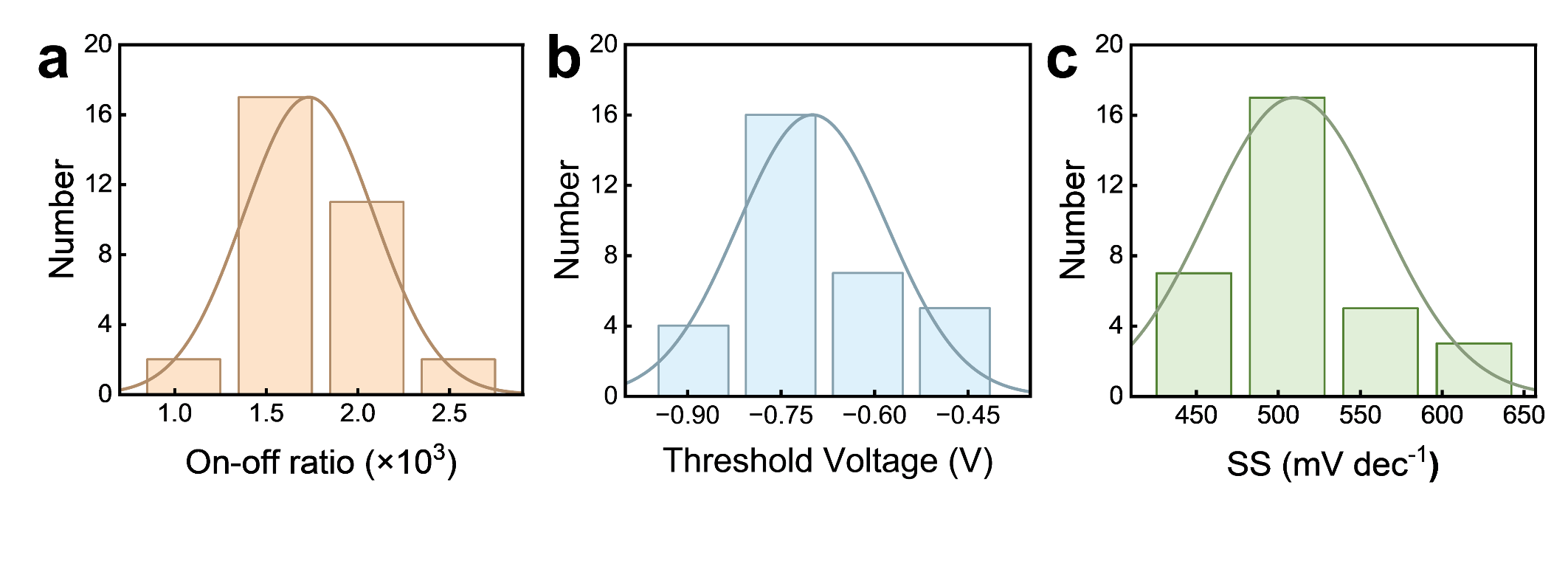


**Figure S13**. Statistical distribution histograms of (a) on-off ratio, (b) threshold voltage (*V_th_*), and (c) subthreshold swing (*SS*) extracted from 32 transfer curves.


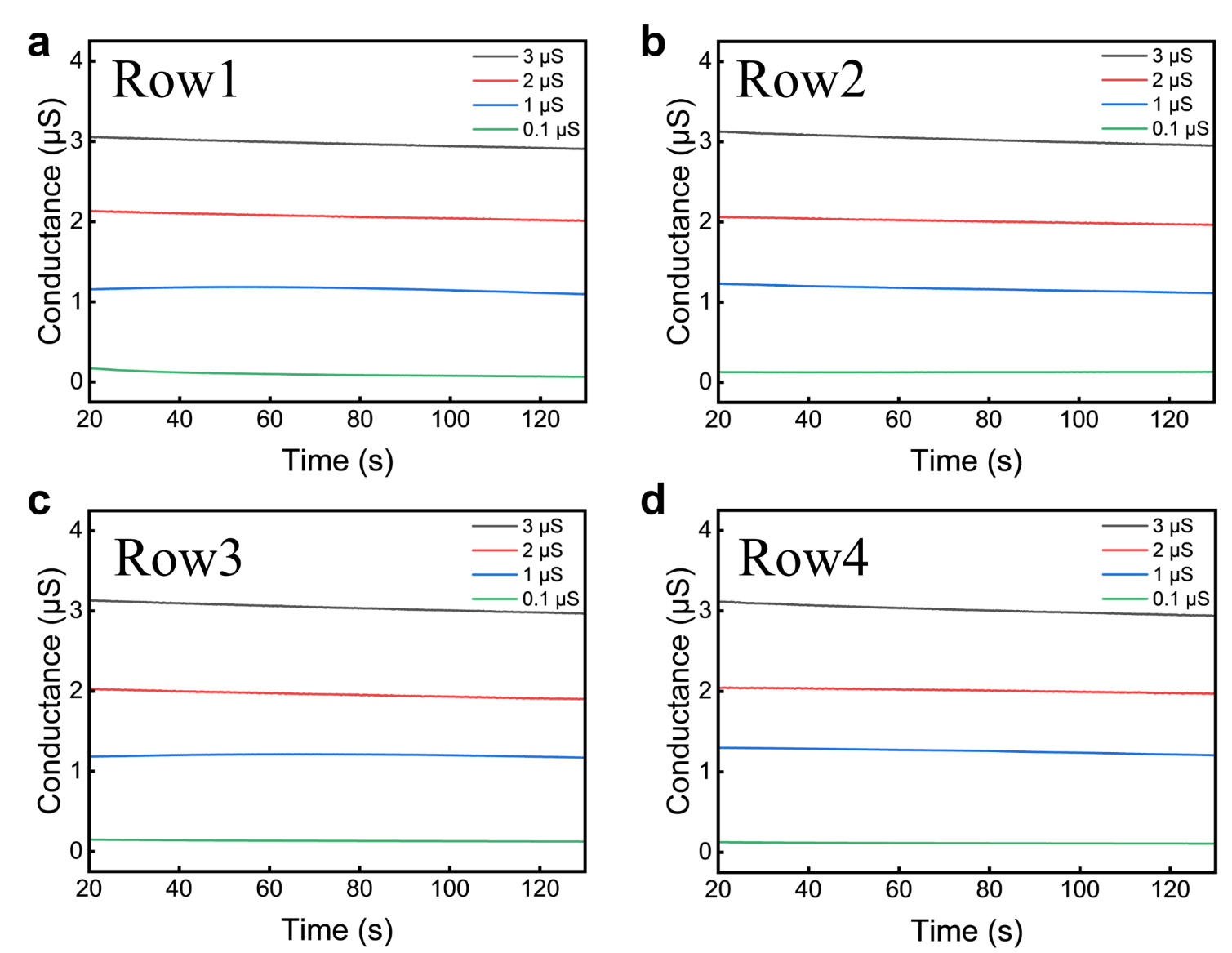


**Figure S14**. Array-level retention characteristics of programmed conductance states. Time-dependent conductance retention of the 4 × 4 P3HT-based EGT array after programming the pixels into four representative conductance levels of approximately 0.1, 1.0, 2.0 and 3.0 μS. (a–d) Retention characteristics of the programmed states in Row 1 to Row 4 measured over 100 s.


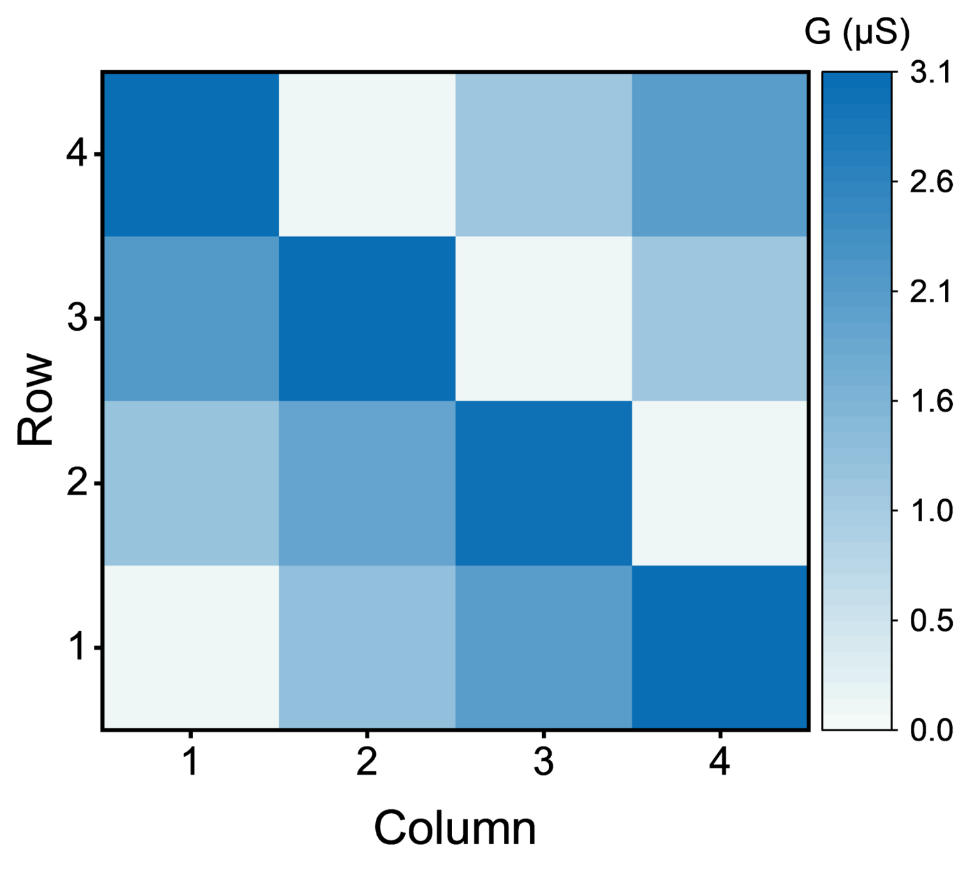


**Figure S15**. Multi-level grayscale conductance programming in the 4 × 4 P3HT-based EGT array. The average conductance of each device was extracted after programming and mapped into a 4 × 4 conductance matrix, showing four distinguishable conductance levels of approximately 0.1, 1.0, 2.0, and 3.0 μS.


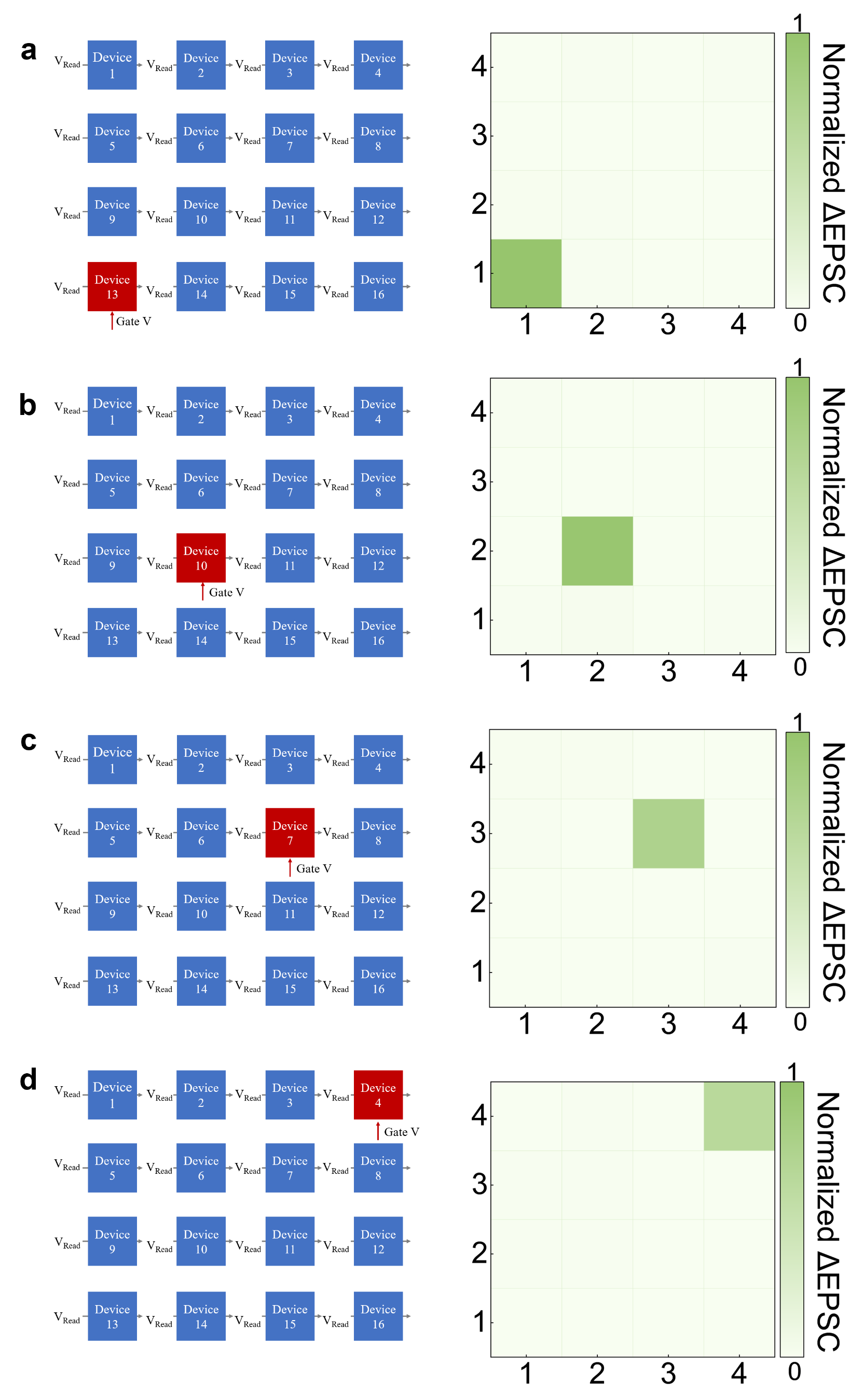


**Figure S16**. Selective-addressing response of the 4 × 4 P3HT-based EGT array. Normalized EPSC maps and corresponding current responses of the array when different pixel units were selectively stimulated by a gate pulse (V_G_ = −3.0 V, 50 ms). The selected pixels show pronounced responses, while the other units remain close to the baseline.

Beyond unit-level uniformity, the array-level performance of the 4 × 4 ionochromic neuromorphic transistor array was further evaluated, including retention stability, multi-level conductance programming, and selective-addressing response. Specifically, the 16 pixel units were programmed into four representative conductance states of approximately 0.1, 1.0, 2.0, and 3.0 μS, which remained clearly distinguishable over 100 s with only slight relaxation and no obvious state overlap (**Figure S**14). Based on the retained conductance values, a 4 × 4 conductance matrix was constructed, confirming multi-level conductance programming at the array level rather than simple binary switching (**Figure S**15). Selective-addressing measurements further show that the target pixel exhibits a pronounced current response under the applied gate pulse (V_G_ = −3 V, 50 ms), whereas the non-addressed pixels remain close to the baseline (**Figure S**16), indicating low inter-pixel crosstalk. These results demonstrate reliable multi-level programming, retention stability, and pixel-level selective operation in the 4 × 4 array, supporting its potential for array-level neuromorphic information storage and visual-state modulation.


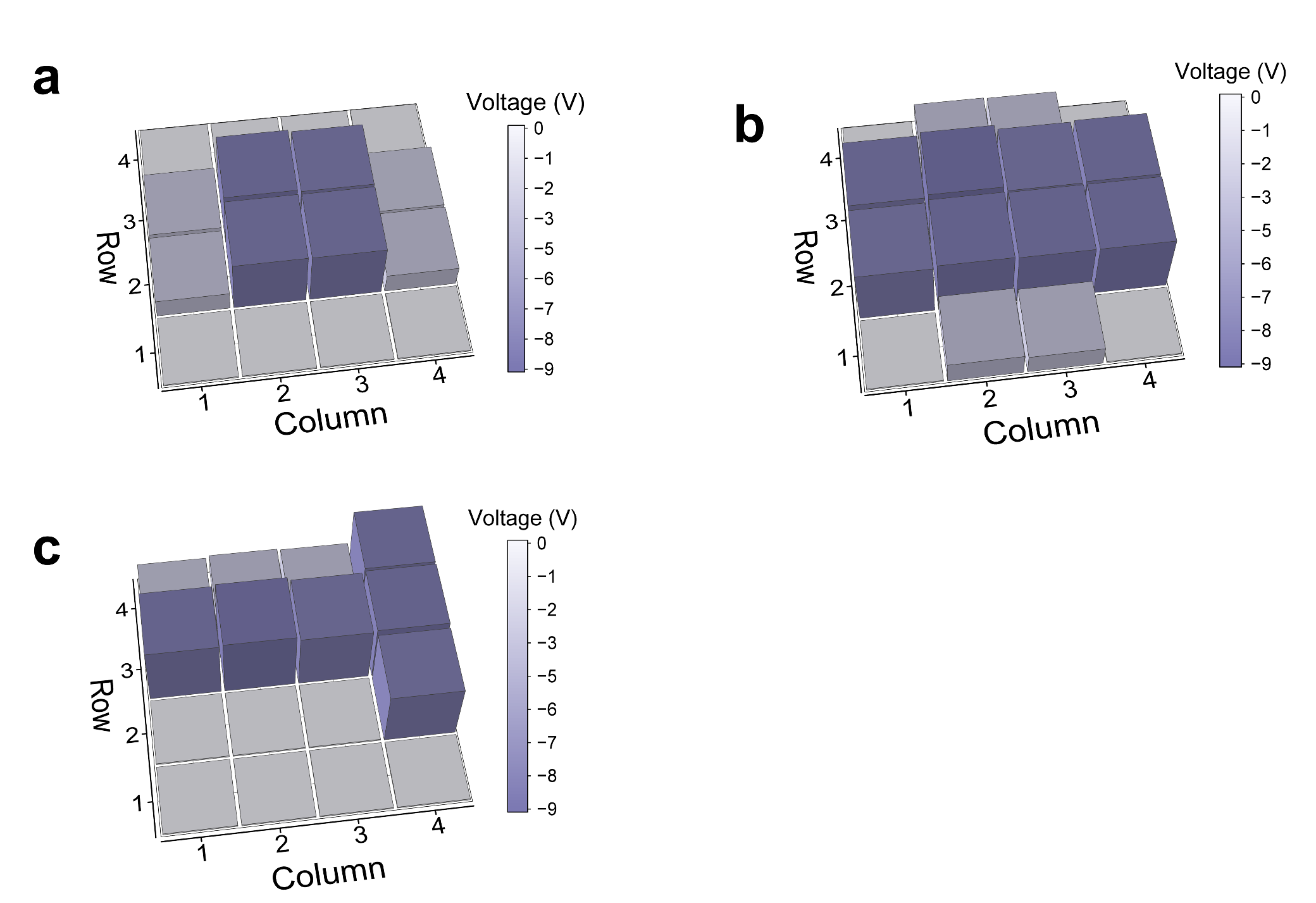


**Figure S17.** Multichannel voltage signals recorded from the sensor array during the grasping of objects with different geometries: (a) a sphere, (b) a cylinder, and (c) a cone.

Supplementary Note 1

The threshold voltage can be extracted by the following equation (1) ^[1,2]^:

$I_{DS}=\frac{WC^{*}D}{2L} \mu{(V_{GS}-V_{th})}^{2}$ (1)

where *I_DS_* stands for the saturated drain-source current. *W*, *L*, and *D* are the width, length, and thickness of the channel, respectively. *C** is the capacitance per unit volume of the channel. *µ* is the saturated mobility. In transfer curves, the intersection of the tangent of the curve with the *V_GS_* axis when *|I_DS_|^1/2^* equals zero was the value of *V_th_*. The *SS* is calculated using the following Equation (2) ^[1][2]^:

$SS=\frac{dV_{GS}}{dLog_{10} I_{DS}}$ (2)

The on/off (*I_on_/I_off_*) ratio is defined as the ratio of the maximum on-state current to the minimum off-state current.

**References**

1. L. Yuan, T. Zhao, J. Dai, et al., “High‐Density, Crosstalk‐Free, Flexible Electrolyte‐Gated Synaptic Transistors Array via All‐Photolithography for Multimodal Neuromorphic Computing,” *Advanced Functional Materials* 35 (2025): 2418052, https://doi.org/10.1002/adfm.202418052.

2. X. Liu, S. Dai, W. Zhao, et al., “All‐Photolithography Fabrication of Ion‐Gated Flexible Organic Transistor Array for Multimode Neuromorphic Computing,” *Advanced Materials* 36 (2024): 2312473, https://doi.org/10.1002/adma.202312473.
